# Disuse-driven plasticity in the human thalamus and putamen

**DOI:** 10.1016/j.celrep.2025.115570

**Published:** 2025-04-11

**Authors:** Roselyne J. Chauvin, Dillan J. Newbold, Ashley N. Nielsen, Ryland L. Miller, Samuel R. Krimmel, Athanasia Metoki, Anxu Wang, Andrew N. Van, David F. Montez, Scott Marek, Vahdeta Suljic, Noah J. Baden, Nadeshka Ramirez-Perez, Kristen M. Scheidter, Julia S. Monk, Forrest I. Whiting, Babatunde Adeyemo, Jarod L. Roland, Abraham Z. Snyder, Benjamin P. Kay, Marcus E. Raichle, Timothy O. Laumann, Evan M. Gordon, Nico U.F. Dosenbach

**Affiliations:** 1 Department of Neurology, Washington University School of Medicine, St. Louis, MO 63110, USA; 2 Department of Neurology, New York University Grossman School of Medicine, New York, NY 10016, USA; 3 Basque Center on Cognition, Brain and Language, Donostia, Gipuzkoa, Spain; 4 Department of Biomedical Engineering, Washington University in St. Louis, St. Louis, MO 63130, USA; 5 Division of Computation and Data Science, Washington University School of Medicine, St. Louis, MO 63110, USA; 6 Department of Psychiatry, Washington University School of Medicine, St. Louis, MO 63110, USA; 7 Mallinckrodt Institute of Radiology, Washington University School of Medicine, St. Louis, MO 63110, USA; 8 Taylor Family Department of Neurosurgery, Washington University School of Medicine, St. Louis, MO 63110, USA; 9 Department of Psychological and Brain Sciences, Washington University in St. Louis, St Louis, MO 63110, USA; 10 Department of Neuroscience, Washington University School of Medicine, St Louis, MO 63110, USA; 11 Department of Pediatrics, Washington University School of Medicine, St. Louis, MO 63110, USA; 12 Lead contact

## Abstract

Subcortical plasticity has mainly been studied using invasive electrophysiology in animals. Here, we leverage precision functional mapping (PFM) to study motor plasticity in the human subcortex during 2 weeks of upper-extremity immobilization with daily resting-state and motor task fMRI. We found previously that, in the cortex, limb disuse drastically impacts disused primary motor cortex functional connectivity (FC) and is associated with spontaneous fMRI pulses. It remains unknown whether disuse-driven plasticity pulses and FC changes are cortex specific or whether they could also affect movement-critical nodes in the thalamus and striatum. Tailored analysis methods now show spontaneous disuse pulses and FC changes in the dorsal posterior putamen and central thalamus (centromedian [CM], ventral-intermediate [VIM], and ventroposterior-lateral nuclei), representing a motor circuit-wide plasticity phenomenon. The posterior putamen effects suggest plasticity in stimulus-driven habit circuitry. Importantly, thalamic plasticity effects are focal to nuclei used as deep brain stimulation targets for essential tremor/Parkinson’s disease (VIM) and epilepsy/coma (CM).

## In brief

Using a 2-week arm and hand casting paradigm with daily brain fMRIs, Chauvin et al. observe subcortical plasticity in humans. The disused motor circuit shows increased functional connectivity and the emergence of spontaneous activity pulses, not tied to movements, in the central thalamus (VIM, CM, and VPL) and posterior putamen.

## Graphical abstract

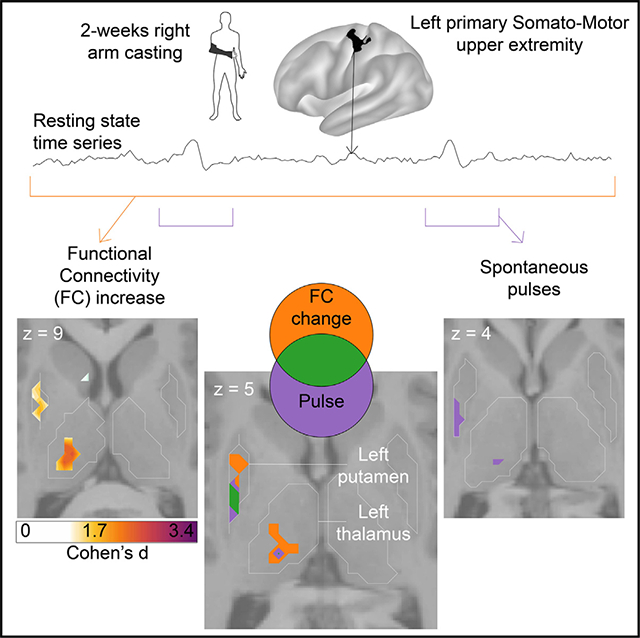

## INTRODUCTION

Brain networks must simultaneously exhibit stability to preserve acquired skills but also flexibility to adapt to environmental changes. Motor behavior is generated by complex cortico-subcortical circuits, including the thalamus, basal ganglia, and cerebellum.^1^ Cortico-striato-thalamo-cortical loops have been studied intensively to understand pathways involved in motor learning. Prior subcortical motor plasticity studies have relied on invasive electrophysiology in patients undergoing deep brain stimulation (DBS)^2,3^ and animal models.^4^ Human neuroimaging studies of motor plasticity have focused on the cerebral cortex owing to the low signal-to-noise ratio of blood-oxygen-level-dependent (BOLD) signals in subcortical structures^5^ and the small effect sizes typically seen in plasticity paradigms.^6^

Motor and action control cannot be understood without consideration of subcortical nodes. Basal ganglia (putamen, caudate, globus pallidus, etc.) and thalamic nodes are intricately networked to facilitate the selection and implementation of intended actions while simultaneously inhibiting conflicting or undesirable ones.^7^ The dorsolateral striatum (posterior putamen and lateral caudate) has been shown to be important for the slow, long-term establishment of stimulus-driven habits, while the dorsomedial striatum (anterior putamen, medial-anterior caudate) is important for goal-directed actions.^1,8^

The central thalamus also plays a significant role in motor learning and control by serving as a relay and integration center between various brain regions involved in motor functions.^9^ The central thalamus comprises multiple distinct nuclei, including the ventral intermediate (VIM), centromedian (CM), and ventroposterior lateral (VPL) nuclei.^10^ The VPL nucleus receives sensory information and relay information for fine-tuning of movement.^11^ The VIM nucleus, in contrast, is primarily involved in planning, initiation, and execution of voluntary movements.^12^ The VIM nucleus is the DBS target in essential tremor and tremor-predominant Parkinson’s disease (PD).^11,13^ A case report from a patient, undergoing DBS, with long-standing bilateral upper extremity loss and use of shoulder-controlled prosthetics suggests plasticity of VIM neurons, leading to an over-representation of shoulder movements.^14^

The CM nucleus and parafascicular nucleus of the thalamus project heavily to the caudate-putamen and nucleus accumbens and more sparsely to the cortex, including the motor cortex,^15^ and therefore might hold a role in sensorimotor function and movement modulation. In the context of studying plasticity, it is worth highlighting that the CM nucleus plays an important role in the regulation of the cortical parvalbumin neurons that promote Hebbian plasticity^16^ and modulate arousal. The CM nucleus is a DBS target in the treatment of refractory generalized epilepsy^17^ owing to its role in sleep-wake regulation and arousal. The central thalamus plays an important role in regulating sleep stages and generates sleep spindles, which play an important role in memory consolidation,^18^ including procedural memory.

To study plasticity mechanisms in the human subcortex, we leveraged an experimental paradigm that induces disuse by restraining the upper extremities using a full-length cast.^19–21^ To obtain sufficient data for within-participant analysis, we casted the dominant (right) upper extremity of three participants (Nico, Ashley, and Omar) for 2 weeks and collected around-the-clock actigraphy and daily task and resting-state functional MRI (fMRI) over a 6- to 8-week experimental protocol (2 weeks pre cast, 2 weeks of casting, and 2–4 weeks post cast). This approach is similar to classic animal plasticity studies, which impose motor or sensory restrictions (e.g., limb constraint, deafferentation, or monocular deprivation) in a small number of intensively studied individuals.^22,23^

Dense longitudinal fMRI sampling enabled us to perform within-participant analyses using our individual-specific precision functional mapping (PFM) methodology.^24,25^ Repeated resting-state functional connectivity (FC) noninvasively maps functional networks within the brain and can reveal how they change in response to disuse. Cortex-only analyses of the casting experiment fMRI data revealed never before observed, spontaneous fMRI pulses in disused motor regions that specifically emerge during the casting period.^19–21^ These disuse pulses were associated with functional disconnection of the left and right primary motor cortex and strengthening of FC between the disused primary motor cortex and cingulo-opercular action-mode network (AMN) regions.^26^ The spontaneous fMRI signal pulses described in the cortex were linked to upper extremity disuse and localized to motor and action control regions (AMN), but many questions remained about their properties and purpose.

Spontaneous pulsatile phenomena detectable with electrophysiological measures, such as thalamic sleep spindles^27^ and pathological subthalamic nucleus beta bursts in PD^28^ arise in the subcortex. Thus, we wondered whether spontaneous disuse pulses detectable with fMRI are purely a cortical phenomenon or whether they occur throughout the entire motor and action system. If spontaneous disuse pulse were to extend to the subcortex, then it would open fascinating lines of inquiry examining whether they might be related to sleep spindles^27^ or the PD beta bursts that correlate with symptom severity.^28^

The discovery of the previously unrecognized somato-cognitive action network (SCAN), which is interposed between effector-specific motor regions along the central sulcus and shows a role in general body movement and movement planning,^29^ has forced a revision of our models of human action and motor circuits, including in the subcortex. This novel reframing of the motor circuit raised the question whether SCAN regions are involved in disuse-driven plasticity and provided further impetus to expand our analyses of disuse-driven plasticity to the whole brain.

However, capturing subcortical changes and comparing sensitivity between the cortex and subcortex are massively complicated by the lower signal-to-noise ratio (SNR) in the subcortex. To overcome the subcortical signal loss, we tailored our analyses to be equally sensitive to plasticity effects in cortex and subcortex using standardized effect sizes (Cohen’s d) and using models of BOLD activity that can adapt better to local signal specificity.

## RESULTS

Casting-driven upper extremity disuse was quantified with actigraphy (Nico, −41%; Ashley, −55%; Omar, −46%).^21^ Following cast removal, participants exhibited reduced grip strength (Nico, 27%; Ashley, 42%; Omar, 39%), which recovered within a week without persistent deficits. At baseline, homotopic motor regions were strongly functionally connected. However, casting-driven right upper extremity disuse caused a marked decrease in effector-specific FC between the left and right motor cortex (r, −0.23 to −0.86) and stronger FC between the disused motor cortex and the AMN.^20,21^

### Disuse strengthens FC of the central thalamus and posterior putamen with the motor cortex

Measurement of FC in the subcortex is challenging, at least partly because of well-known fMRI SNR drops off toward the center of the brain as distance from the receiver coil elements increases^5^ (see Figure S1 for SNR drop-off maps in this dataset) and heterogeneity in cerebral blood flow across the brain.^30,31^ Consequently, the magnitude of FC changes between different conditions (e.g., Cast-Pre) are greater in the cortex than in the subcortex. Therefore, to measure FC changes in the subcortex, we used standardized effect size (Cohen’s d) to account for the signal-to-noise differences. The present effects, expressed in terms of Cohen’s d, match our previously reported parcel-based FC findings.^20^ Disuse-driven FC increases (Cohen’s d) of left SM1_ue_ were significant within AMN regions (Figure S2, purple border), perimotor cortex (Figure S3), and hand motor regions of the cerebellum (Figure S3) for all three participants, with Ashley generally showing the strongest effects. Importantly, disuse-driven left SM1 FC changes spared adjacent regions of the SCAN (Figure S2, maroon outlines), a set of effector-general action control regions in the motor cortex^29^ that are strongly functionally connected to the AMN (see also Figure S3). Thus, the two SCAN regions adjacent to upper extremity-specific primary motor regions (black seed region, left SM1_ue_) did not show significant FC changes with casting, highlighting a sharp division of functional changes specific to the effector-specific motor cortex.

**Figure 1.**
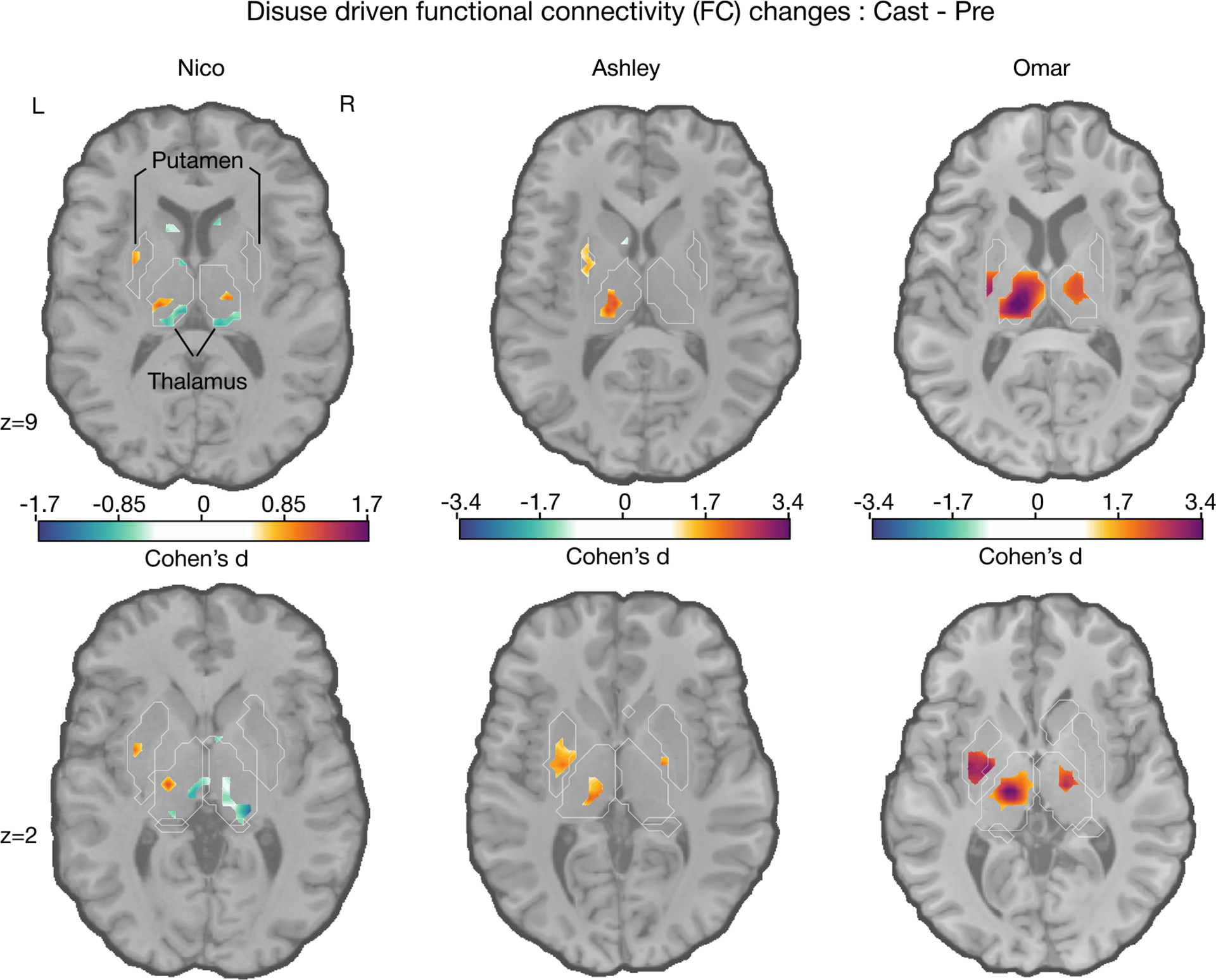
Disuse-driven changes in FC of the left effector-specific primary somatomotor cortex to the subcortex For each participant (left to right: Nico, Ashley, and Omar), individual-specific plasticity effect size (Cohen’s d) maps show changes in left SM1_ue_ FC during right (dominant) arm casting (pre cast). For reference, a Cohen’s d of 0.8 is generally considered a large effect size. Only significant effects after cluster correction at *p* < 0.05 (STAR Methods) are displayed. Note that Nico’s data were collected using an earlier pulse sequence with a repetition time (TR) that was twice as long (2.2 s) compared to that used for Ashley and Omar (1.1 s). Nico’s effect sizes are about half the size of those of the other participants. The FreeSurfer-based anatomical borders of the putamen and thalamus are shown as white outlines.

The Cohen’s d reveals large effects on subcortical structure. In the subcortex, all three participants showed statistically significant increases in FC between disused left SM1_ue_ and the central thalamus as well as the posterior putamen (Figure 1, bottom row; cluster-based thresholding; STAR Methods; Table 1). Cortical effects (Cohen’s d) were large, while relatively smaller effects were observed in the subcortex and the cerebellum (Table 2).

Other FC changes were significant in some but not all participants. In Ashley and Omar, an area with significant FC increases was observed bilaterally in the left posterior globus pallidus. In Omar, an increase FC in the right central thalamus was also observed. However, in Nico, FC decreases were observed in the right central thalamus and the most posterior part of the left and right thalamus. In Ashley, an FC decrease in the caudate nucleus was observed. Ashley’s and Omar’s effect sizes were roughly twice those observed in Nico. These differences may be attributable to fMRI pulse sequence differences (repetition time [TR] 1.1 s Ashley and Omar vs. 2.2 s Nico), differences in fMRI SNRs, or inter-individual differences (baseline FC differences or behavioral adaptation strategies).

In the cortex, disuse-driven FC changes reverted back to baseline within 2 weeks after cast removal.^20^ Therefore, we tested for significant FC changes after removing the cast (Post - Cast). Consistent with the cortex, the same subcortical regions that had shown FC effects of casting-driven disuse (Figure 1) also showed a reversal of those effects after cast removal (Figure S4). The plasticity time courses in the cortex and subcortex were very similar (Figure S5; for additional slices through the caudate nucleus, see Figure S6).

### Disuse pulses in the central thalamus and motor cerebellum

We previously reported the emergence of large spontaneous fMRI signal pulses in the disused motor cortex after 12–48 h of arm casting.^21^ These disuse pulses were absent at baseline and disappeared again after the cast was removed. They were also specific to the disused hemisphere, with only a few observed in the right hemisphere (SM1_ue_) during casting, not enough for properly powered analyses.

**Figure 2.**
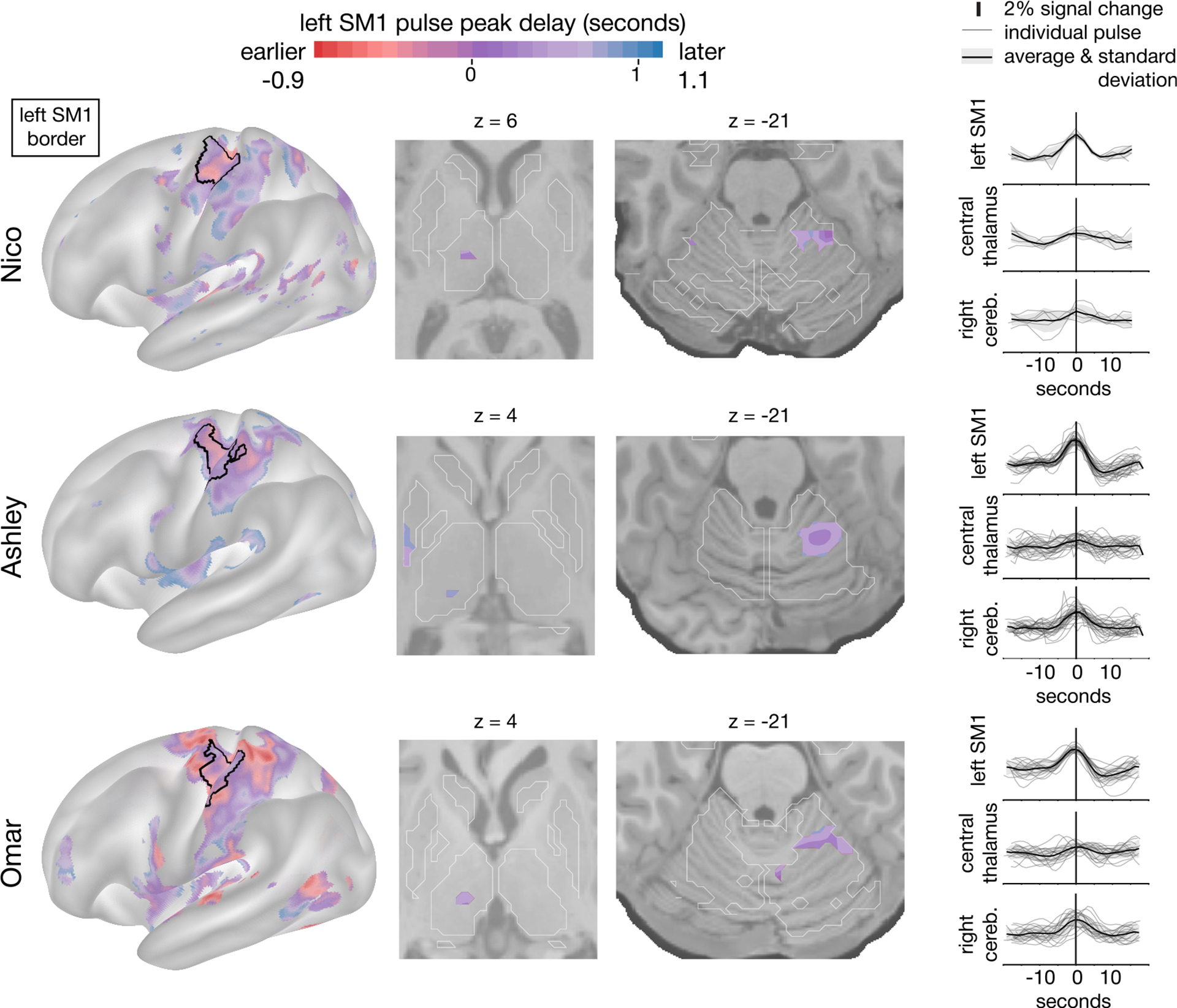
Disuse pulse distribution in the cortex and subcortex Spontaneous pulses detected in each of the participants (Nico, top; Ashley, center; Omar, bottom) are shown on an inflated rendering of the left cortical hemisphere (left) and subcortical axial slices (center, thalamic and cerebellar views). The color scale spans 2 s bracketing the average left SM1_ue_ pulse peak. The maps display the voxels with highest pulse detectability (top 20 %; STAR Methods). The participant-specific upper extremity somatomotor region is outlined in black (left). On the right, the individual (thin lines) and average (thick line) pulse time courses are shown (y axes, percent signal change) for the left SM1_ue_, the left thalamus, and right cerebellum. The time course of each disuse pulse was modeled using a hemodynamic response function (HRF) (STAR Methods).

To identify the time delay of disuse pulses in the subcortex, we developed an hemodynamic response function (HRF)-based pulse detection method that allows flexibility in HRF shape and timing compared to the event cue (here, the time of cortical SM1 pulses) (STAR Methods). Using this HRF-based detection method, we confirmed their spatial distribution in the cortex (Figure 2, left column) and cerebellum (Figure 2, third column)^21^ and were able to detect the presence of disuse pulses in the subcortex, which was not detected with our previous approach. Disuse pulses in the central thalamus were observed in all participants (Figure 2, second column). The peak pulse percent signal change in the thalamus was lower compared to the cortex (Figure 2, right column). In the participant with the most disuse pulses overall (Ashley), they were also present in the posterior putamen. The least number of pulses were detected in Nico, who also exhibited relatively weaker FC changes.

Disuse pulses propagate through the brain in a specific temporal sequence. We previously reported that SMA regions peak earlier than left SM1_ue_, followed by the cerebellum.^21^ Here, on average, the central thalamus pulses peaked later than in left SM1_ue_ (Figure 2, first and second columns; Nico, +0.75 s [SD 0.16]; Ashley, +1.07 s [SD 0.02]; Omar, +0.95 s [SD 0.09]).

Pulse detection was successfully replicated using a standard double gamma HRF (Figure S7) and finite impulse response (FIR) (Figures S8 and S9). Directly comparing the cortical and subcortical pulse peak amplitude, full width half-maximum (FWHM) and time delay for the HRF and FIR analyses revealed that they provide the same results (Figures S10 and S11).

### FC changes and disuse pulses overlap in the central thalamus

**Figure 3.**
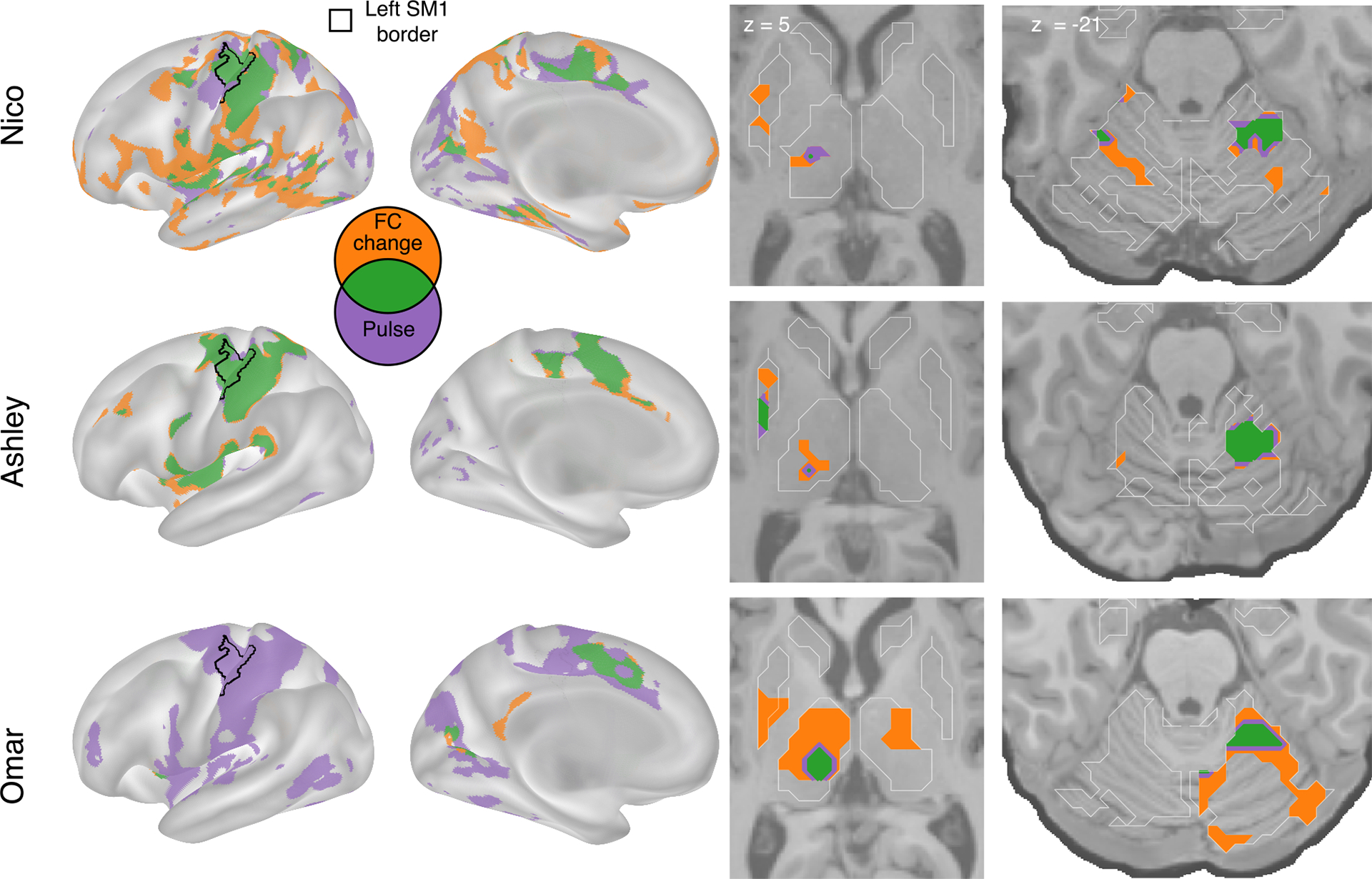
Spatial overlap of FC increases and disuse pulses The strongest disuse-driven FC increases (orange, cast > pre, cluster corrected) and disuse pulses (purple, top 20% threshold) as well as their overlap (green) are shown on the cortical surface (left) and in the thalamus and putamen (center) and cerebellum (right). Results are displayed on the lateral left hemisphere surface, medial left hemisphere surface, and two axial slices (MNI z = 5 and −21). White borders on axial slices define individual specific FreeSurfer-based anatomical structures (z = 5: putamen, globus pallidus, caudate, and thalamus; z = −21: cerebellum and hippocampus).

Figure 3 illustrates the topography of pulses in relation to FC changes. All participants showed overlap (green) between disuse-driven FC changes and pulses in the dorsal medial cortex (SMA, pre-SMA, and dorsal anterior cingulate cortex), the central thalamus, and the effector-specific motor regions of the cerebellum (Figures 3 and S3). Ashley’s pulses in the posterior putamen also overlapped with FC increases.

### Subcortical disuse-driven plasticity overlaps with fMRI task activations

**Figure 4.**
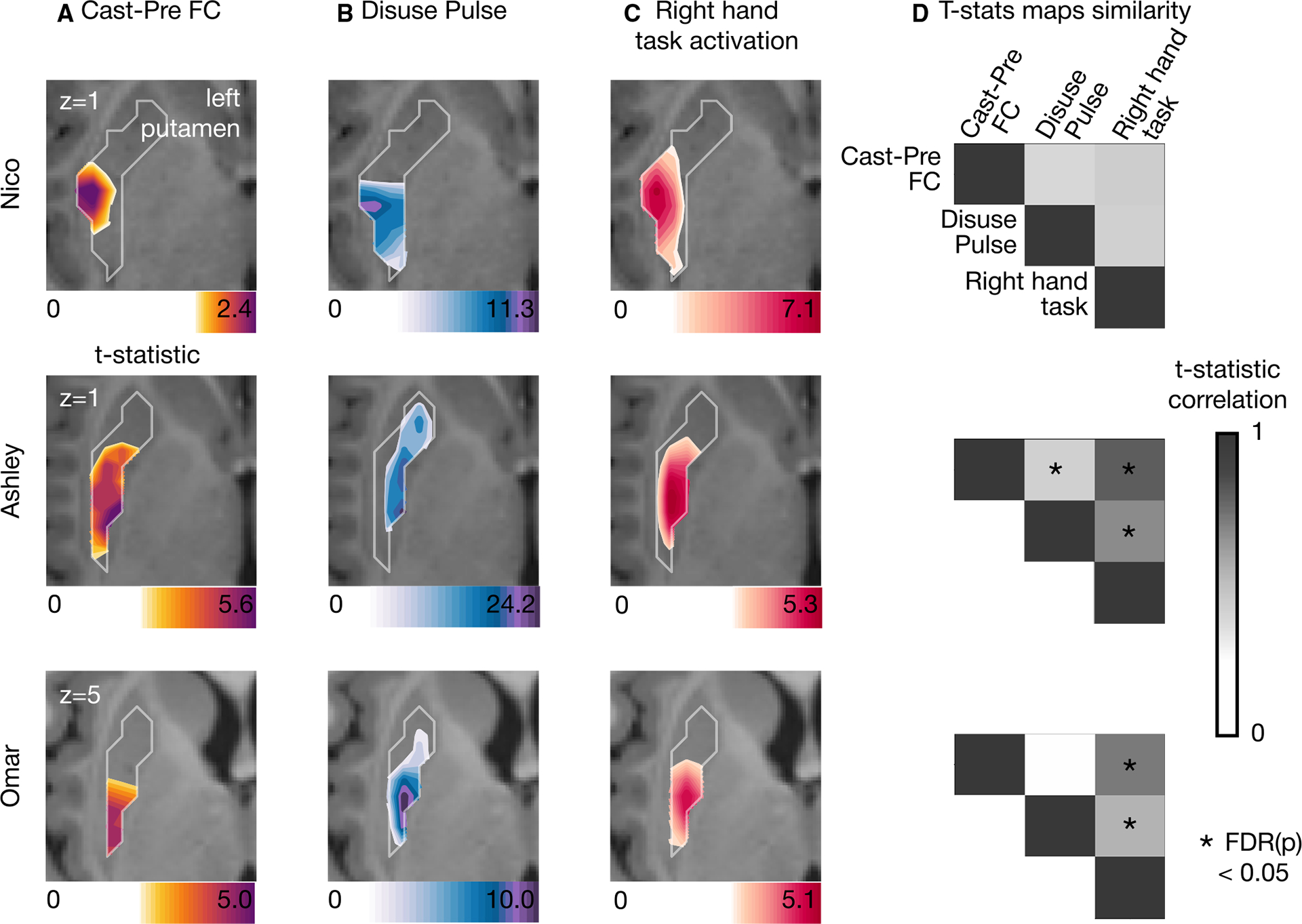
Putaminal disuse-driven FC changes, pulses, and hand movement task fMRI activations (A) Map of disuse-driven increases in FC with the left SM1_ue_ region of interest (top 30 % t-statistics). (B) Map of disuse pulses (top 30 % t-statistics). (C) Map of pre-casting task fMRI contrast: right hand movement vs. baseline (top 30% t-statistics). For all three maps, color scales are represented at the bottom of the map with maximum value at 99.5%. (D) Correlation between left putamen t-statistic maps for disuse-driven FC increases, pulse, and activation during hand movement (right hand vs. baseline). Correlations between unthresholded t-statistic maps were tested against individual-specific null distribution effects for each participant (top to bottom: Nico, Ashley, and Omar). Reported significant *p* < 0.05 corrected for false discovery rate (FDR; black asterisks).

**Figure 5.**
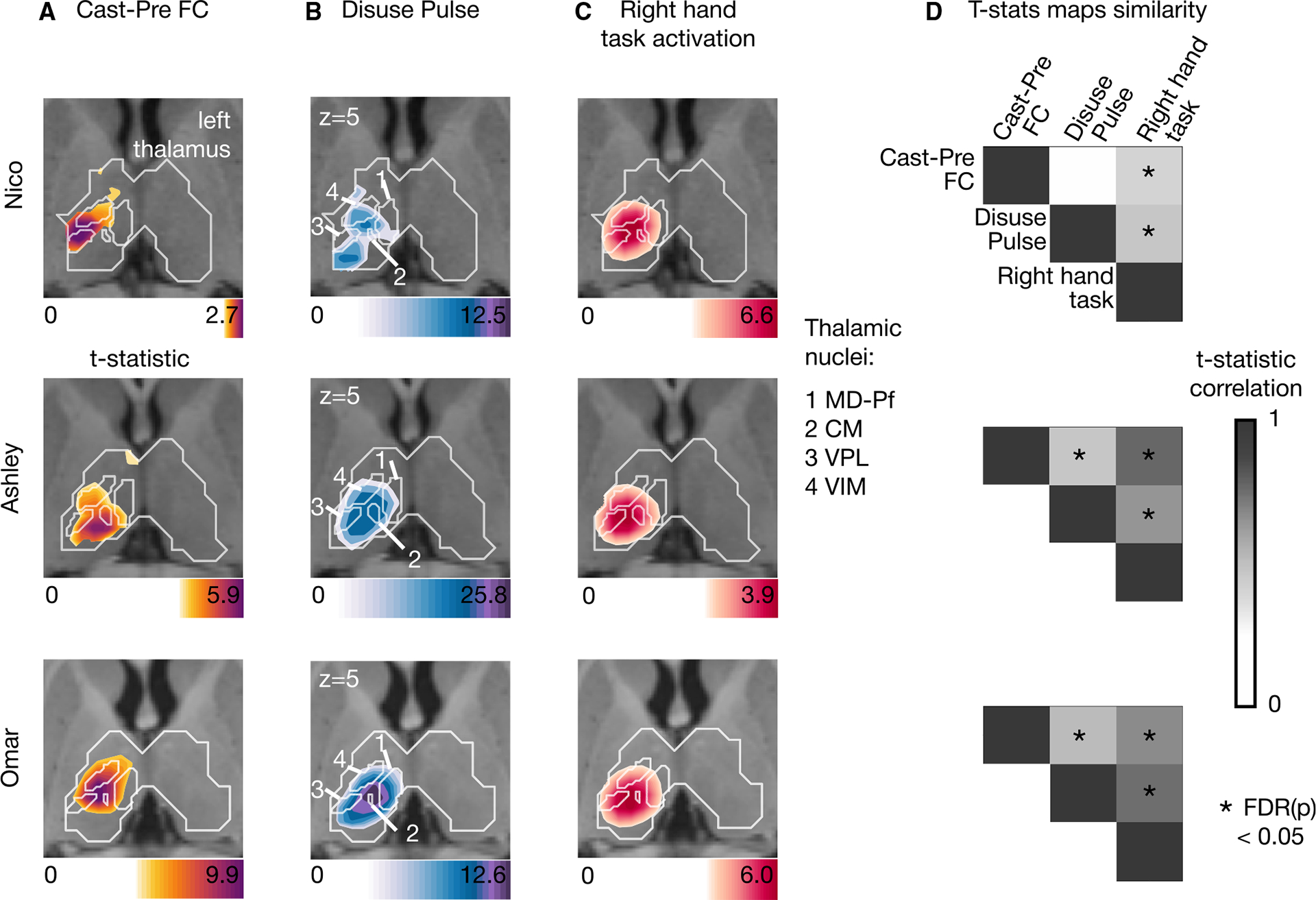
Thalamic disuse-driven FC changes, pulses, and hand movement task fMRI activations (A) Map of disuse-driven increases in FC with left SM1_ue_ region of interest (top 30% t-statistics). (B) Map of disuse pulses (top 30% t-statistics). (C) Map of pre-casting task fMRI contrast: right hand movement vs. baseline (top 30% t-statistics). For all three maps, color scales are represented at the bottom of the map with maximum value at 99.5%. Nucleus borders from the Thalamus Optimized Multi Atlas Segmentation (THOMAS) atlas individual-based segmentation overlapping with the effects are shown in white. Four nuclei are outlined: CM (centromedian), VPL (ventro-posterior lateral), VIM (ventral intermediate), and MD-Pf (medio-dorsal). Average t-statistics values for all thalamic nuclei are represented in bar plots (Figure S12). (D) Correlation between left thalamic t-statistic maps for disuse-driven FC increases, pulses, and activations during hand movement (right hand vs. baseline). Correlations between unthresholded t-statistic maps were tested against individual-specific null distribution effects for each participant (top to bottom: Nico, Ashley, and Omar). Reported significant *p* < 0.05 corrected for FDR (black asterisks).

To determine whether FC changes and disuse pulses spatially coincide with regions active during upper extremity movement, we analyzed previously unreported motor task fMRI data collected at baseline (Figures 4 and 5; see Figure S13 for non-normalized, unthresholded maps). The motor task includes simple hand, tongue, and foot movements administered in a block design^32^ (STAR Methods). This paradigm elicited somatotopically specific responses in the primary motor cortex (Figures S14 and S15).

All three participants showed motor task fMRI activation in the posterior putamen and central thalamus in response to right hand movement (Figure 4). In each participant, task activations overlapped with loci of FC change and disuse pulses. This similarity was confirmed by the strength of correlation between t-statistic maps. Ashley and Omar showed strong and significant topographic similarities between motor task responses and both FC change and pulse density maps. For Nico, the participant with the lowest SNR, the overlap was less clear (Figure S13). This indicates that task-activated circuits, and the two disuse-driven plasticity markers, pulses, and FC change, overlap in the thalamus and putamen given sufficient signal strength. The statistical significance of map similarity (Figures 4D and 5D) was assessed using individual-specific null distributions (STAR Methods).

To delineate the specific thalamic distribution of cast-induced plasticity effects in comparison to upper extremity motor circuitry, we quantified the average t-statistic values within each thalamic nucleus from the THOMAS atlas (Figures 5 and S12; for individual THOMAS atlas segmentation, see STAR Methods^10^). The thalamic nuclei showing the most significant plasticity effects were the CM, VPL, and VIM nuclei (see Figure S16 for all participants). Individual-specific effects can be noted. One participant (Omar) also showed additional strong activation in motor task response, FC change, and pulse density maps for the medio-dorsal parafascicular nucleus, and Nico also showed pulses in the medial geniculate nucleus. However, right hand movement was associated with consistent activation of the CM, VPL, and VIM nuclei across all participants. This set of nuclei remained specific when quantifying t-statistic average values from right to left hand movement task contrast, thus confirming the specificity of the right hand movement circuitry (Figure S14). The CM, VPL, and VIM nuclei all showed disuse pulse as well as disuse-induced FC changes with left SM1_ue_.

## DISCUSSION

### Disuse driven plasticity effects are not limited to the cerebral cortex

We observed significant subcortical FC changes and disuse pulses in the central thalamus (overlapping VIM and CM nuclei) and posterior putamen as a result of dominant upper extremity disuse, imposed by casting. These findings suggest that disuse can drive network changes at all levels of motor circuitry: cortex, cerebellum, thalamus, and striatum. In the cortex, the SCAN regions adjacent to the disused effector-specific primary motor region did not show an increase in FC or pulses, which highlights the functional differentiation between SCAN and effector-specific motor networks. Throughout motor and action control circuits, as verified by task fMRI activation, FC increases partially overlapped with the presence of disuse pulses, suggesting that they might represent a brain-wide plasticity mechanism. We previously demonstrated that the presence of disuse pulse does not fully explain the changes in FC.^20^ Pulses and FC changes overlap for two of three participants. The presence of disuse pulses in the thalamus suggests a more general phenomenon that likely acts in concert with other neuronal and synaptic plasticity mechanisms.

### Disuse strengthens FC in motor and action-mode subcircuitry

Increased FC between subcortical and cortical motor regions in the presence of pulses is consistent with our previously reported finding of strengthened FC between the motor cortex and the AMN.^20^ However, these results cannot be fully accounted for by a simple Hebbian-like process,^33^ which should weaken FC in the setting of disuse (reduced firing together). Instead, we speculate that pulses, by creating co-activation in the disused motor control circuit, could increase within network synchronization, thus playing a role in plasticity to help maintain the integrity of disused subcircuits.^21^ Pulses in the disused subcircuits may enable rapid recovery of both behavior (actigraphy) and FC (within days) after cast removal.^21^

Prior work in animal models suggests that neural plasticity in response to changes in auditory or visual stimuli starts with a reduction of inhibitory interneuron activity at the population level, increasing excitatory activity, which facilitates Hebbian plasticity.^16,34^ While the fMRI signal cannot differentiate specific inhibitory/excitatory population activity,^35,36^ we can hypothesize that the observed FC increase within the motor execution circuit could represent increased simultaneous firing with consequent Hebbian plasticity and information retention. Local disinhibition could account for the emergence of spontaneous activity pulses. Hypothetically, such pulses may play a physiological role in plasticity processes or in opening windows for cortico-thalamic information exchange.

### Disuse-driven plasticity affects the posterior putamen, involved in motor habit formation

Rapid, goal-directed learning is thought to primarily involve the dorsomedial striatum, including the caudate nucleus, whereas slower acquisition of habits, which are insensitive to changes in the reward value of the outcome, is thought to depend more on the dorsolateral striatum, including the posterior putamen.^37^ The increased FC with the posterior putamen may be a correlate of processes that protect existing motor skills or processes that facilitate the acquisition of new skills. Indeed, on the first day following cast removal, our participants kept using their non-casted arm more than their recently freed dominant arm even though fine motor coordination was unimpaired, suggesting that participants briefly persisted in casting-induced movement patterns even after the cast was removed. Pre-casting movement patterns resumed on day 2 after cast removal.^21^

### Plasticity in thalamic nuclei for motor execution

The central thalamus is important for motor and action execution. Studies have tied movement performance to activity in the VIM, VPL, and CM thalamic nuclei,^3^ which is consistent with our motor task fMRI activation (Figures S14 and S15). The VIM nucleus is part of the thalamic ventrolateral region, primarily involved in motor control and relay of information between the basal ganglia, cerebellum, and motor cortex. In contrast, the VPL nucleus is part of the somatosensory system that relays touch, temperature, and pain information from the body to the primary somatosensory cortex.^38^ The VIM nucleus is an effective DBS target for tremor. In cases of neurological injury or disease, such as stroke or neurodegenerative disorders, the VIM can undergo plasticity as part of the brain’s adaptation and recovery mechanisms.^14^ In cats, for example, VIM nucleus stroke results in specific skilled locomotor impairment (e.g., impaired ladder mobility but intact flat walk).^39^ Reorganization of activity in the VIM nucleus after the stroke correlates with restored mobility.^40,41^ Following partial VIM nucleus lesions, intricate motor behavior, such as walking on a complex surface, returns within a few days, demonstrating the highly effective plasticity of the thalamus.^39^ While the thalamus is essential for cortical organization and specialization during development,^42^ plastic reorganization of specific motor programs can also occur in response to motor demands, driving functional adaptation in adults. For example, in humans, in the context of amputation, one study observed changes in the representation of the affected body parts within the VIM nucleus. Electrophysiological recording in a patient with arm amputation and long-term use of a prosthetic device demonstrated an enlargement of the shoulder representation in the VIM, which is now used for prosthetic grip control by the patient.^14^

### Thalamic plasticity pulses and sleep spindles

In addition to the VIM and VPL nuclei, the CM nucleus also showed FC increases, pulses, and task fMRI activation. The CM nucleus plays a special role in regulating arousal.^15^ While the VIM nucleus is the DBS target of choice for treatment of tremor, the CM nucleus is targeted in the treatment of Tourette syndrome and, with increasing frequency, epilepsy and disorders of consciousness.^17^

Indeed, the thalamus has a specific role in regulating sleep pressure and wakefulness.^43^ The thalamus regulates sleep stages and slow oscillations in deep sleep.^18^ Sleep events such as thalamo-cortical spindles are thought to facilitate consolidation of episodic as well as procedural memory.^27^ During deep sleep, slow waves of activity are observed across the cortex and relayed in the thalamus.^43^ These slow waves are thought to facilitate homeostasis of neural activity after a day of novel experiences.^33^ The slow waves increase synchronization and phase-locked communication between regions. Slow waves are phase locked to other sleep phenomena, like thalamic sleep spindles, and their synchrony is a predictor of sleep-dependent memory consolidation. Thalamic sleep spindles occur in the beta frequency band and promote plasticity in the motor network.^44^

An earlier study from Huber et al. showed that, following just 12 h of immobilization, motor-evoked potentials were decreased, and overnight sleep electroencephalograms (EEGs) showed reduced sleep slow-wave and sleep spindle activity in sensorimotor regions.^45^ In our study, disuse pulses in the awake fMRI signal only began to emerge after 24–48 h of immobilization. Prolonged immobilization studies that combine awake and asleep EEG and fMRI are needed to better understand how length of disuse, state (awake vs. asleep), and signal type (EEG vs. fMRI) relate to each other.

The occurrence of disuse pulses in the central thalamus, with the CM showing consistent high t-statistic values during pulse activity (Figure S13), nonetheless raises the question whether they might be phenomena similar to thalamocortical sleep spindles. Could the disuse pulses represent a circuit-specific, sleep-like phenomenon occurring during the awake resting state? Indeed, local sleep can be observed during wakefulness.^46^ After a long period in an awake state, EEG recordings in awake rats can capture local “offline” states similar to sleep, together with slow waves.^47^ As slow waves are temporally phased with thalamo-cortical sleep spindles, we can hypothesize that they can also occur during the awake state. While this local sleep has only been shown in animals, it shows that these sleep plasticity phenomena are not only available during sleep. Therefore, plasticity pulses could be the first observation of awake thalamocortical sleep spindle-like events. Consistent with this idea, Ashley and Omar, who were imaged in the evening, after a day of novel cast-related experience, showed more pulses than Nico, who was scanned in the morning. However, with only three participants, this difference in number of pulses could also be explained by other differences between participants. This insight is worthy of follow-up investigation.

### Cortico-striato-thalamic FC increase as a marker of disuse in PD

In our upper extremity immobilization paradigm, we observed increases in FC in the disused motor circuit. Importantly, these FC changes rapidly reversed following cast removal.^20^ In PD, a slowly progressive neurodegenerative disease, action impairments include tremor, rigidity, and bradykinesia as well as general paucity and slowness of movement. These deficits follow progressive loss of dopaminergic neurons projecting to the striatum.^2^ Similar to casting-induced FC changes, patients with PD show a maladaptive increase in FC between the putamen, central thalamus, and cortical motor area, especially during akinesia.^48,49^ This suggests a potential pathophysiological link between paucity of action and PD. One hypothesis might be that increased FC in motor and action circuits may reflect increased neuronal excitability during behavioral idling.^50^

The VIM nucleus is a target for DBS treatment of tremor. Electrophysiological studies in patients with PD and parkinsonian mouse models have revealed increased beta power and prolongation of beta burst discharges propagating throughout cortico-basal ganglia circuits.^51^ These beta bursts disappear with movement or L-dopa treatment.^28^ Further research is needed to determine whether beta bursts detected with electrophysiology in parkinsonism could show some similar disuse-related pulses.

### Plasticity and stability in the subcortex

Disuse-driven FC changes and spontaneous activity pulses are not confined to the cortex but extend into the putamen and central thalamus (VIM, CM, and VPL nuclei). This anatomical pattern, especially the prominence in the central thalamus, suggests parallels to sleep-related mechanisms of consolidation and plasticity as well as to the beta bursts seen in PD. These findings open intriguing new avenues for studying disorders such as PD and normal sleep physiology. They also raise the interesting possibility that the mechanisms for changing the brain and for maintaining it are one and the same.

### Limitations of the study

The repeated sampling with PFM and intervention design enabled us to observe large effects across participants but also revealed significant participant-specific effects. It remains unclear whether inter-individual differences were due to idiosyn-crasies in plasticity mechanisms or distinct behavioral adaptation strategies or whether we were insufficiently powered to capture the same effects in all participants. Some inter-individual variability could also be explained by differences in the fMRI sequence. The fMRI pulse sequence used for Nico had a TR twice as long (2.2 s) than that used for Ashley and Omar. This difference is also visible in the lower SNR for Nico’s data.

In this study, we selected analytical methods optimized for subcortical signal characteristics. However, the original dataset was acquired to capture plasticity effects at the whole-brain level; thus, the fMRI sequence itself was not optimized for the subcortex. We chose a 4.7 mm FWHM smoothing kernel,^52,53^ based on prior work, but this smoothing might have limited the significance of the thalamic nucleus specificity analyses (Figure S12).

## STAR★METHODS

### EXPERIMENTAL MODEL AND STUDY PARTICIPANT DETAILS

#### Human participants

We analyzed a previously published dataset that comprised three healthy, right-handed adult volunteers (Nico, 35 years old, male; Ashley, 25 y.o., female; Omar, 27 y.o., male). All participants were right-handed, as assessed by the Edinburgh Handedness Inventory^56^ (Nico: +100, right handed; Ashley: +91, right-handed; Omar: +60, right-handed. The Washington University School of Medicine Institutional Review Board approved the study protocol and provided experimental oversight. Participants provided informed consent for all aspects of the study and were paid for their participation.

#### Experimental setup

Arm immobilization was conducted by constraining the participant’s dominant (right) arm for two weeks (cast period). The immobilization followed a two-week experimental baseline (pre-cast period) and was followed by a recovery period of two weeks (post-cast period). For one participant (Nico), the pre-cast period was acquired one month before the cast period and data was consistently acquired at 5 AM, while for the two other participants, fMRI was acquired at 9 PM. For one participant (Omar), the cast was removed and reapplied after one day of the cast period to adjust for finger comfort. Details of cast construction are described in Newbold et al.^21^

#### Imaging data

On each day of the experiment, a scan session was conducted to acquire structural and functional data. Structural MRI was consisted of four T1-weighted images (sagittal acquisition, 0.8 mm isotropic resolution, 3D MP-RAGE, Gradient echo) and four T2-weigthed images (sagittal acquisition, 0.8 mm isotropic resolution, 3D T2-SPC, Spin echo). A 30-minute resting state fMRI (rs-fMRI) run was acquired during each session. Two runs of the HCP Motor strip mapping task^32,57^ were acquired for each pre- and post-cast session. Ashley and Omar’s fMRI was acquired with an improved fMRI sequence (all 2D Gradient echo, echo planar, TR: 1.1 vs. 2.2 seconds in Nico, 2.6 vs. 4 mm isotropic resolution in Nico). All data were resampled at 3mm in atlas space. Acquisition parameters and procedures are detailed in Newbold et al.^21^

### METHOD DETAILS

#### Precision functional analysis

All data processing and analysis was conducted at the participant level using individually defined functional and anatomical boundaries. All statistical testing was done against null distributions built for each participant.

#### MR image processing

Preprocessing of structural and functional images, denoising of rs-fMRI data, and cortical surface projection were performed as previously described^21^ and involved temporal interpolation to correct for differences in slice acquisition timing, rigid-body correction of head movements, correction for susceptibility inhomogeneity-related distortions, and alignment to atlas space. The present results are reported in MNI152 space. FreeSurfer version 5.3^54^ was used for anatomical segmentation and labeling of amygdala, accumbens, caudate, cerebellum, hippocampus, pallidum, putamen and thalamus, on left and right hemisphere. Subcortical regions were not eroded, we instead performed null testing with spatial constraint (see quantification and testing of effects against individual null distributions) to account for statistical significance by anatomical structure. There were no systematic differences in head movement (mean FD or number of frames removed) between phases of the casting protocol.

The rs-fMRI data denoising involved removal of high-motion frames (framewise displacement [FD] > 0.1 mm), band-pass filtering (0.005–0.1 Hz), and regression of nuisance time series, including head movement parameters, the global signal averaged across all gray-matter voxels, and orthogonalized waveforms extracted from ventricles, white matter, and extracranial tissues.

The rs-fMRI was then projected to cortical surface as the last step. The cortical surface was smoothed using a two-dimensional 6-mm full-width half-maximum (FWHM) smoothing kernel. The volumetric data for the smaller subcortical and cerebellar structures were smoothed less, using a three-dimensional 4.7-mm FWHM kernel.

For two analyses (Hemodynamic response function modeling of the pulse spatiotemporal representation analyses using rs-fMRI and for the motor system localizer HCP task data^32^), denoising of data involved high-pass filtering (0.1Hz) instead of band-pass filtering as low pass would affect the model fitting. Smoothing was performed after the modeling.

Fully processed data are available in the Derivatives folder of the Cast-Induced Plasticity dataset in OpenNeuro (https://openneuro.org/datasets/ds002766).

### QUANTIFICATION AND STATISTICAL ANALYSIS

#### Signal-to-noise ratio

Signal-to-noise ratio (SNR) is calculated on the data before noise regression as the average signal overtime divided by variance of signal overtime for each run. Maps provided in Figure S1 are the average SNR map across all fMRI rest runs, transformed to percent by multiplying by 100 and displayed as signal drop (in percent) by removing the maximal value from the map.

#### Primary somatomotor upper extremity region of interest

The upper extremity SM1 region was defined in individual using a task-based approach (top 1th activation) combined with automatic labeling by FreeSurfer. Details were previously described in Newbold et al.^21^

#### Individual network representation

A set of 18 canonical functional networks was defined for each participant using a graph theory-based community detection algorithm with anatomical priors^25^ (see Figure S1) (infomap, https://www.mapequation.org/). This algorithm assigns grayordinates to communities. Subsequently, these communities are categorized based on their similarity to established group-average networks, recently updated to include the SCAN.^29^

#### Thalamic nuclei segmentation using THOMAS

We employed the Thalamus-Optimized Multi-Atlas Segmentation (THOMAS v 2.1) method, a promising approach for identifying nuclei,^10^ using the hips_thomas.csh function. The choice of THOMAS followed a recent consensus of nuclei naming and automatic segmentation algorithm improvement.^58,59^ We also aimed to interpret our results in relation to clinical applications and needed a robust localization of VIM, which has been shown to co-localized with the segment labeled VLPv (Ventro-Lateral-Posterior ventral) using the THOMAS segmentation.^13^

This version has been validated exclusively for T1-weighted acquisitions and is accessible through Docker (https://github.com/thalamicseg/thomas_new). The average T1-weighted image, generated for the registration of all functional data, was employed for this purpose. Nuclei were mapped into cifti format at the resolution of functional images (3mm^3^) to quantify overlap between brain maps and thalamic nuclei. Voxels were assigned by highest overlap with the T1 resolution when transformed to functional resolution. Spatial null testing (see quantification and testing of effects against individual null distributions) ensures specificity of results for each substructure by controlling for spatial spill-over due to the spatial resolution and smoothing.

#### rs-fMRI functional connectivity (FC)

BOLD fMRI time series were analyzed for each individual specific vertices/voxels (i.e., cifti grayordinates) to obtain full brain FC for each rs-fMRI session. Seed-based FC was estimated as the average of FC maps for each voxel in the seed. Pre-cast FC maps were averaged over sessions to define the baseline FC.

#### rs-fMRI FC change

For casting sessions comparison to baseline (pre-cast) sessions, Cohen’s d was calculated at each grayordinate. Cohen’s d indexes change in FC effect size relative to standard deviation. This allows higher sensitivity to subcortical casting effects as the subcortical correlation values are lower in comparison to cerebral cortex but consistent across sessions. Cohen’s d is also more stable than the t-statistic to variation in the number of measurements between participants or between casting protocol phases. Due to repeated measure design, the sample size is small (only 14 sessions per phase). Thus, loss of a single session impacts the interpretation of t-statistic more than Cohen’s d. Statistical significance was assessed within participants using individually generated nulls. To define significant effects, we computed null distribution FC maps by randomizing session labels 1000 times and computing a multi threshold cluster-based correction. 10 thresholds were defined within the range of values of the original data, using values of every 10^%^ of the values distribution. Cluster of data passing cluster size correction at p<0.05 for at least two thresholds are displayed in Figure 1. Cluster size correction was estimated for each anatomical structure (cortical and subcortical) independently.

#### Pulse detection and modeling

High amplitude fMRI signal pulses were previously described in cortex as a specific casting-related event. In depth analysis, including using a pressure bladder to detect small hand movement in the cast, demonstrated that pulses were a spontaneous neural event unrelated to motor behavior. These proof points hold for the current investigation of the presence of pulse activity in other regions that the cortex. To detect “pulses”, e.g., in Left SM1_ue,_ we look for a lateralized variation of fMRI activity exceeding a standard level of activity. Thus, the average BOLD time series for the Left and Right SM1_ue_ were computed for each rs-fMRI run and variance normalized across all runs. The difference between Left and Right time series was computed; pulses were detected at time points exceeding 2.3 times the standard deviation of this difference and with an increase of signal in Left SM1_ue_ time series above 2.3 standard deviation. To avoid real movement event detection as a pulse, as a head movement can create a variation in signal that can detected as a pulse but visibly does not show the shape of a pulse, automatically detected pulses with a high correlation (>0.8) with head movement (on a 18s-window centered on the pulse peak) were removed to eliminate contamination from head motion. We show in Figure S17 that movement (Framewise displacement, FD) is not elevated surrounding pulse windows.

In order to study the subcortical pattern of the disuse pulse described in Newbold et al.,^21^ we used a pulse detection analysis sensitive to potential differences in hemodynamic response function (HRF) shape in subcortical regions. At each grayordinate, we modeled a pulse waveform using the HRF shape at each pulse (double gamma HRF function available in nipy (SPM based hrf double gamma, nipy.modalities.fmri.hrf) and optimized using scipy (scipy.optimize.curve_fit)). Model convergence within the parameter range characterized the presence of pulse activity. Regions with the most frequent pulse activity (20% highest pulse detection) are displayed in Figure 2. Pulse latency at each pulse locus was computed as the temporal difference between peaks relative to Left SM1_ue_. The displayed map was thresholded at 1.1 seconds (TR) after the Left SM1_ue_ pulse peak.

To compare results across methods, we provide standard (fixed parameters) double gamma HRF maps, resulting from FSL feat^60^ in Figure S7.

We also performed a Finite Impulse Response (FIR) analysis using a TENT basic function from afni^61,62^ 3dREMLfit and 3dDeconvolve to verify that the findings hold with a method that does not assume a specific BOLD model. We analyzed a window of 33 seconds (17.6 seconds before the peak of the pulse detected in the Left SM1ue regions and 15.4 seconds after). As this method requires higher statistical power for a good time series estimate, we were only able to obtain a time series at the resolution of the TR (2.2 s for Nico and 1.1 s for Ashley and Omar). The resulting time series for each voxel was used to calculate the peak height, full width half maximum and peak delay. The peak amplitude was the maximal value at each voxel in the FIR window, the full width half maximum is the time difference between the time point at half amplitude of the max values, and the peak delay was the time of peak relative to the peak time in Left SM1ue.

To compare these maps with the nipy HRF parameter, we extracted the peak_disp parameter, the standard deviation of the Gamma distribution and that is linked to the FWHM by a factor of 2*√ (2 *ln (2)).

For the amplitude, to compare output from both analysis, we normalize the distribution adjusting for non-gaussianity using mixture modeling to capture the main Gaussian mean.

#### Motor task analysis

Task block designs for each movement condition (Tongue, Left Hand, Right Hand, Left Foot, Right Foot) were modeled using a double gamma HRF in a GLM analysis from FSL feat.^32^ Block onset and offset were modeled as independent events.^63^ Analysis was conducted independently on surface and subcortical grayordinates, following the HCP pipeline steps (release v4.3). Second level analysis across runs within participants was performed with FSL and the resulting t-scores were used to study thalamic responses.

#### VIM localization

Voxels representing VIM were determined using an indirect approach based on anterior commissure - posterior commissure (ACPC) formula, as we do not have specific T1 contrast to visualize nuclei borders. We used the Guiot’s relationships to localize the VIM^64^ that estimate 0.25 length of the ventricle from the PC point and a lateral coordinate equal to the sum of one-half the width of the third ventricle. Review of the literature showed variation in the formula. We considered voxels in sagittal plane between 14 mm from the center of the third ventricle and 11 mm from the border of the third ventricle.^13^ We considered voxels 3mm superior and 2mm anterior to these points that represent the size of the VIM. To this end, the T1-weighted image was aligned on the ACPC line using ACPC detect.^65^ We used the FreeSurfer segmentation of the third ventricle to estimate VIM coordinates. Voxels within the three axis ranges were identified at a resolution of 3mm.

#### Quantification and testing of effects against individual null distributions

The significance of results in each anatomical region (e.g., subcortical structure or thalamic nuclei) was assessed relative to a null distribution (1000 random whole brain representations), taking into account the spatial autocorrelation property of the fMRI signal, using Moran spectral randomization that account for spatial constraint.^66^ The null distribution was constructed by structure to account for local specificity of the signal and relative resolution and size of structure. Tests were corrected for multiple comparisons across participants using false discovery theory.

#### Temporal dynamic model

For left putamen, left thalamus and right cerebellum and the caudate (Figures S5 and S6), regions of interest for the temporal dynamic were selected as the regions of significant change in FC during casting compared to pre casting, using 2-cluster thresholding, overlapping with the top 1% motor execution activity per structure (as defined by our motor task (left hand vs baseline). This selection allowed us to capture the dynamic of regions of the motor executive circuit and differentiate them from regions with other functional roles. We modeled an exponential decay with two alphas as described in Newbold et al.,^21^ using a least-squares approach for each participant.

## Supplementary Material

1

## Figures and Tables

**Table 1.** Disuse-driven FC changes in the subcortex

	Nico	Ashley	Omar
Left posterior putamen	−31 -6 0	−25 -12 0	−31 -9 3
	N_vox_ = 43	N_vox_ = 76	N_vox_ = 71
Left thalamus	−16 -21 6	−7 -21 0	−13 -24 6
	N_vox_ = 68	N_vox_ = 72	N_vox_ = 219

Shown are peak coordinates (x, y, and z) in Montreal Neurological Institute (MNI) space and cluster size (N_vox_) for disuse-driven increases in FC with left SM1_ue_. Clusters are in the left putamen and left thalamus.

**Table 2.** Disuse-driven FC change amplitude

	Nico	Ashley	Omar
Left posterior putamen	1.17	2.56	3.09
Left thalamus	1.24	2.83	3.86
Cerebellum	2.22	2.68	4.79
Cortex	2.43	4.01	4.49

Shown are maximum Cohen’s d values of disuse-driven increases in FC with left SM1_ue_ in significant clusters of the left putamen, left thalamus, cortex, and cerebellum.

**Table:** KEY RESOURCES TABLE

REAGENT or RESOURCE	SOURCE	IDENTIFIER
Deposited data		
Raw and processed MRI data	Newbold et al.^21^	OpenNeuro: ds002766; https://openneuro.org/datasets/ds002766
Software and algorithms		
Python	Python Software Foundation	RRID: SCR_008394; https://www.python.org/
Freesurfer	Fischl^54^	RRID: SCR_001847; https://surfer.nmr.mgh.harvard.edu/
Connectome Workbench	Marcus et al.^55^	RRID: SCR_008750; https://www.humanconnectome.org/software/connectome-workbench
Analysis code	This manuscript	https://doi.org/10.5281/zenodo.14975944; https://github.com/roscha/subcortical-cast
NITRC	NITRC-R	RRID: SCR_003430; http://www.nitrc.org/
4dfp tools	N/A	ftp://imaging.wustl.edu/pub/raichlab/4dfp_tools/

## Data Availability

Further information and requests for materials and data should be directed to and will be fulfilled by the lead contact, Roselyne Chauvin (chauvin@wustl.edu). This study did not generate new materials. The neuroimaging data used in this study have been previously deposited in OpenNeuro (dataset ID ds002766, https://openneuro.org/datasets/ds002766) and are publicly available as of the date of publication. Accession numbers are listed in the key resources table. All original code generated in the analysis of this study has been deposited online at Zenodo.com (https://doi.org/10.5281/zenodo.14975944) and the related GitHub repository (https://github.com/roscha/subcortical-cast) and is publicly available as of the date of publication. Accession numbers are listed in the key resources table. Any additional information required to reanalyze the data reported in this work paper is available from the lead contact upon request.

## Highlights

- Limb disuse increases functional connectivity (FC) within cortico-subcortical motor circuit
- Limb disuse causes spontaneous activity pulses in corresponding motor cortex and subcortex
- Central thalamus and posterior putamen show disuse pulses and increased FC
- Thalamic plasticity is centered on VIM, CM, and VLP nuclei
