# Supplementary material for: Disuse-driven plasticity in the human thalamus and putamen
Source: Cell Rep. Author manuscript; Available in PMC 2025 May 29. (PMC12120925; doi:10.1016/j.celrep.2025.115570)
Supplement: 1 [file NIHMS2076724-supplement-1.pdf]

Cell Reports, Volume *44*

Supplemental information

Disuse-driven plasticity

in the human thalamus and putamen

Roselyne J. Chauvin, Dillan J. Newbold, Ashley N. Nielsen, Ryland L. Miller, Samuel R. Krimmel, Athanasia Metoki, Anxu Wang, Andrew N. Van, David F. Montez, Scott Marek, Vahdeta Suljic, Noah J. Baden, Nadeshka Ramirez-Perez, Kristen M. Scheidter, Julia S. Monk, Forrest I. Whiting, Babatunde Adeyemo, Jarod L. Roland, Abraham Z. Snyder, Benjamin P. Kay, Marcus E. Raichle, Timothy O. Laumann, Evan M. Gordon, and Nico U.F. Dosenbach

Supplemental Information

Supplemental figures and tables

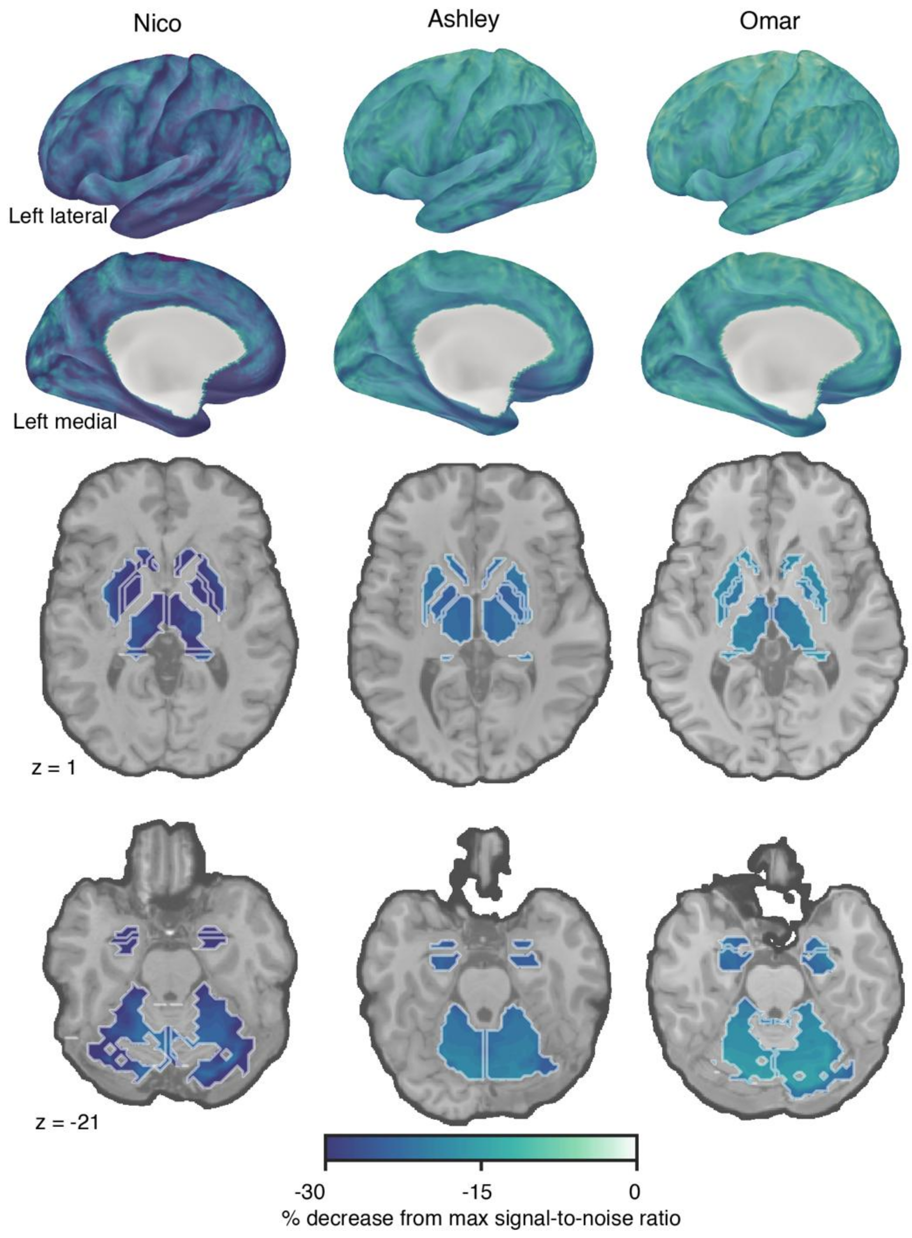

Figure S1: Signal-to-noise ratio (SNR) maps. *Percent SNR decreases from the maximum value are displayed for the cortical left lateral and medial view and subcortical transverse slices (z = 1 and -21). The Freesurfer based anatomical border of cerebellum, putamen, pallidum, thalamus, hippocampus and caudate are overlaid on the axial slices.*

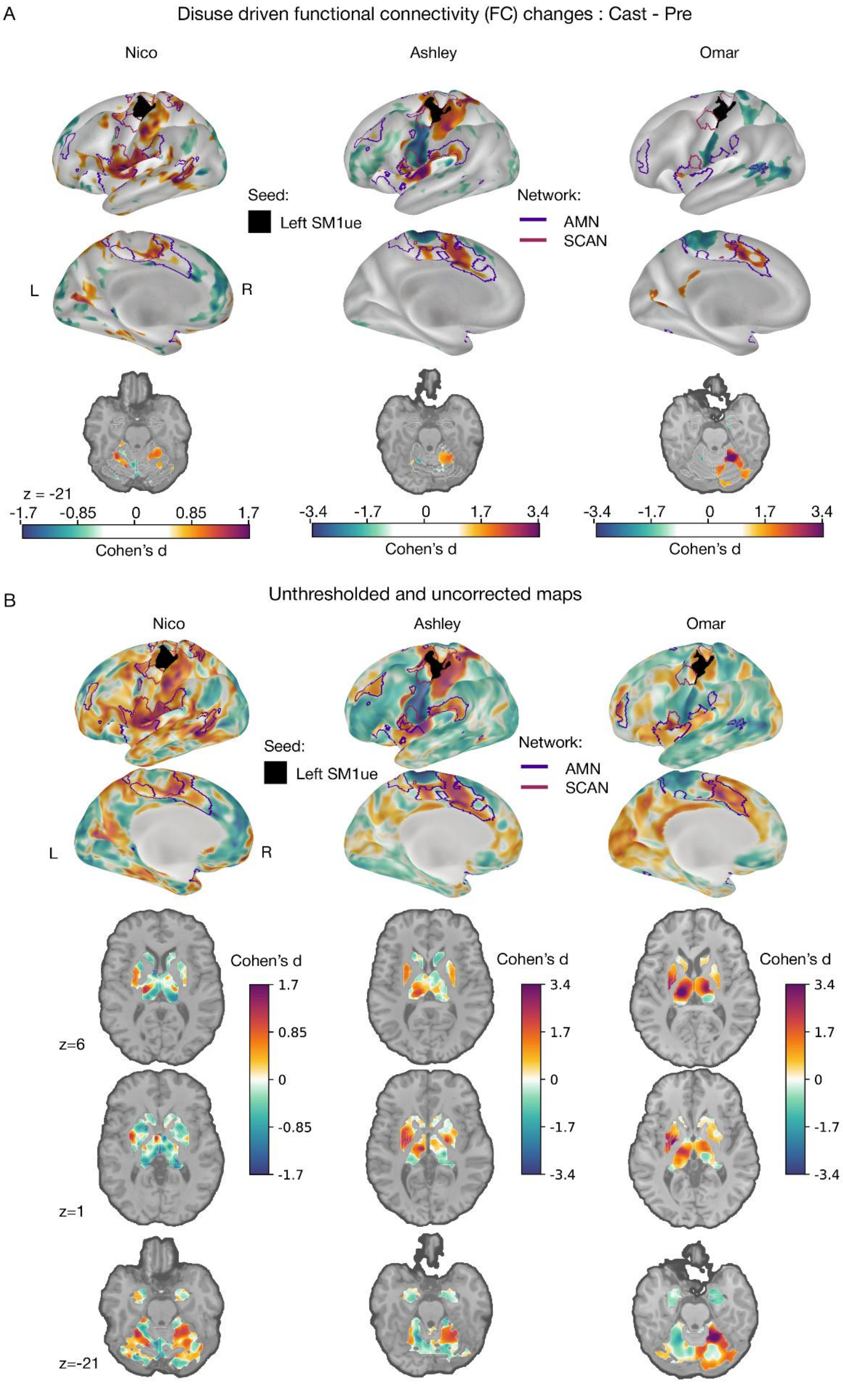
A
Disuse driven functional connectivity (FC) changes : Cast - Pre
Nico
Ashley
Omar
Seed:
Left SM1ue
Network:
AMN
SCAN
L
R
z = -21
-1.7
-0.85
0
0.85
1.7
Cohen's d
-3.4
-1.7
0
1.7
3.4
Cohen's d
B
Unthresholded and uncorrected maps
z=6
z=1
z=-21

Figure S2: Disuse-driven changes in functional connectivity (FC) of effector-specific primary somatomotor cortex (left $SM1_{ue}$) in cortex and cerebellum. *Individual-specific plasticity effect size (Cohen's d) maps showing changes in FC during casting (Cast − Pre) for the left $SM1_{ue}$ (black), and for each participant (left to right columns: Nico, Ashley, Omar). A) after cluster testing B) unthresholded, uncorrected maps. For reference, a Cohen's d of 0.8 is generally considered a large effect size. Only significant effects after cluster correction at $p < 0.05$ (see Methods) are displayed. With a TR that is twice as long, Nico's effect sizes are about half the size of the other participants. The functional network borders of the action-mode (AMN, purple) and somato-cognitive action (SCAN, maroon) networks are displayed on the inflated surface rendering (top row). The Freesurfer based anatomical border of cerebellum is overlaid on the axial slices (bottom row).*

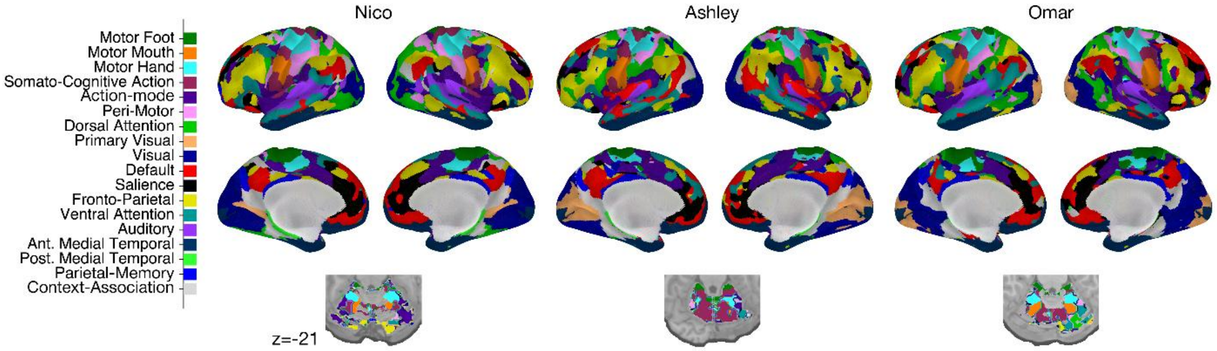

Figure S3: Individual-specific functional networks *Individual-specific definition of 18 canonical networks using the Infomap algorithm (Nico, top; Ashley, middle; Omar, bottom) Inflated cortical view on left and cerebellum on right.*

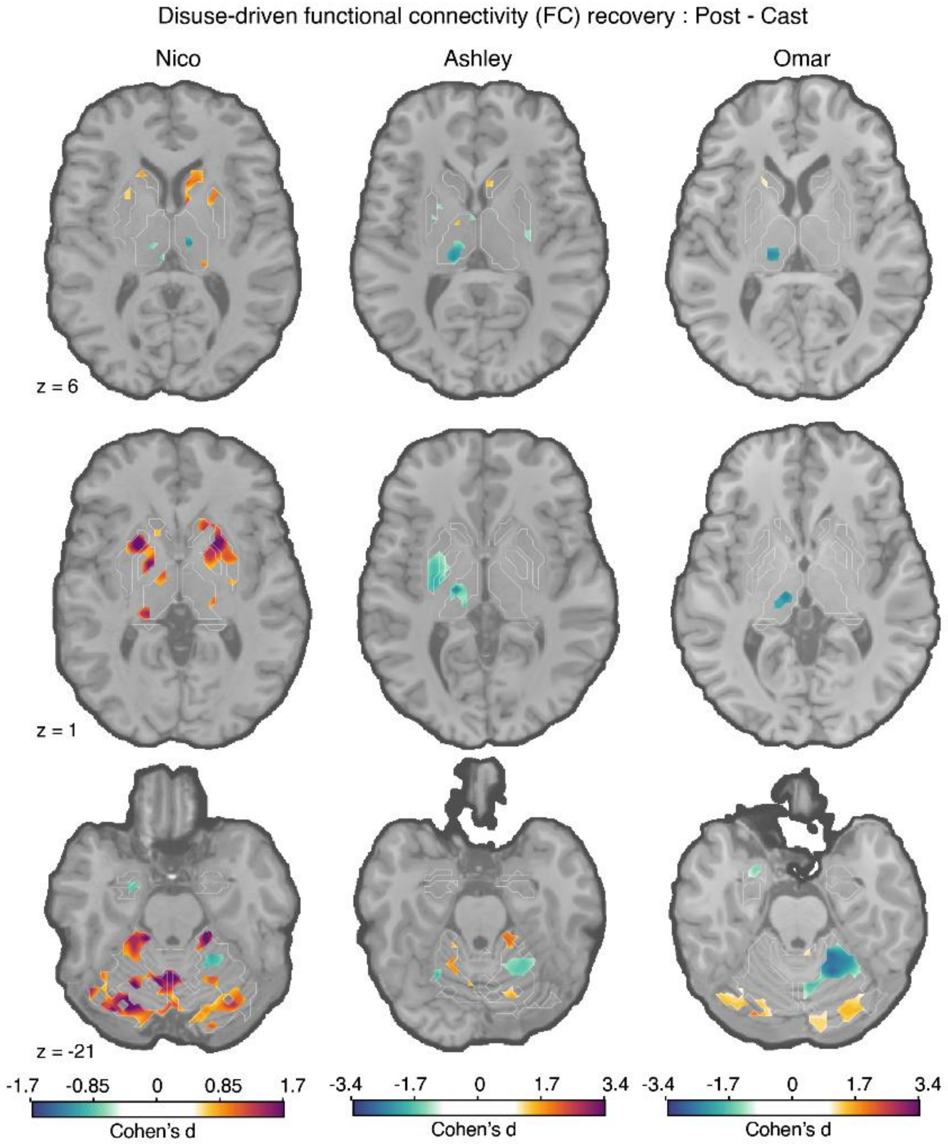

Figure S4: Disuse-driven recovery of functional connectivity (FC) of the left effector-specific primary somatomotor cortex (left $SM1_{ue}$) in subcortex. *Individual-specific plasticity effect size (Cohen's d) maps showing changes in left $SM1_{ue}$ FC after right arm casting removal (Post – Cast) for each participant (left to right columns: Nico, Ashley, Omar). For reference, a Cohen's d of 0.8 is generally considered a large effect size. Only significant effects after cluster correction at $p < 0.05$ (see Methods) are displayed. Please note, Nico's data were collected using an earlier pulse sequence with a TR that was twice as long (2.2 s) compared to Ashley and Omar (1.1s). Nico's effect sizes are about half the size of the other participants. The Freesurfer based anatomical border of cerebellum, putamen, pallidum, thalamus, hippocampus and caudate are overlaid on the axial slices (z= -21, 1 and 6).*

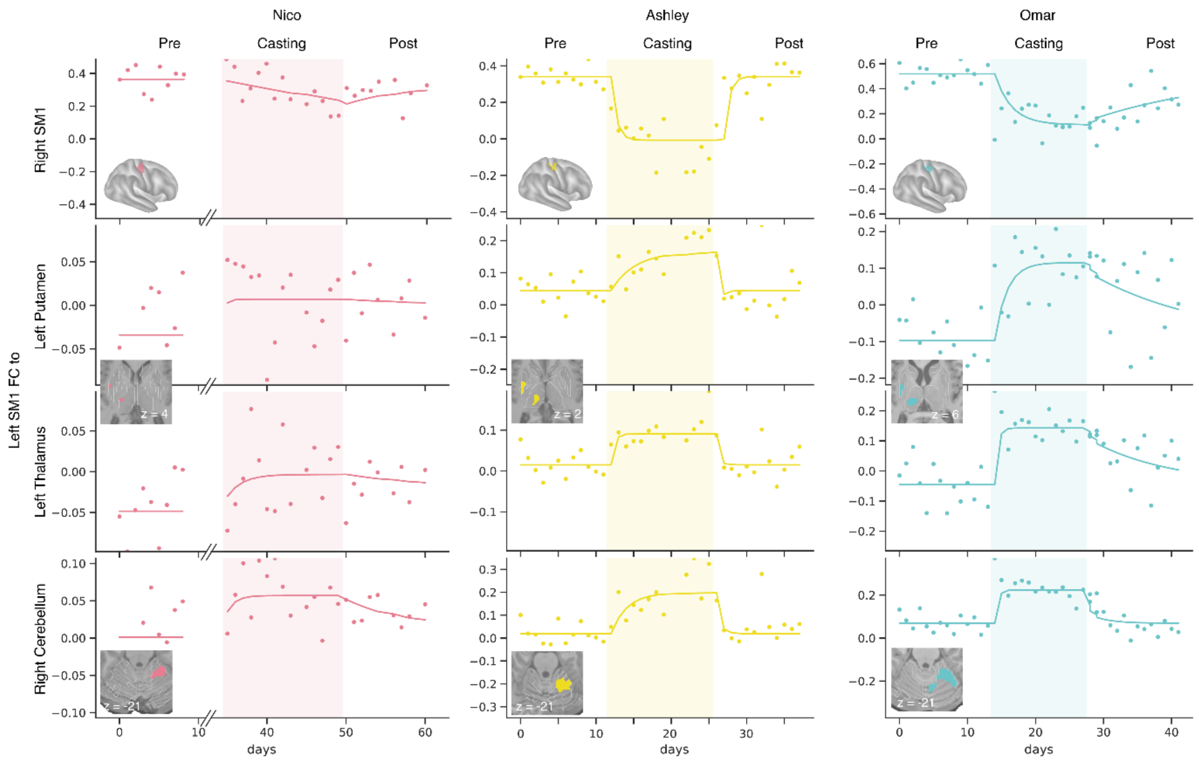

Figure S5: Left SM1$_{ue}$ functional connectivity (FC) timecourses across the casting protocol. *FC time course over the casting protocol between left SM1$_{ue}$ and right SM1, left putamen, left thalamus and right cerebellum. Regions of interest are selected as the voxels of significant increase after cast removal that overlap with the top 1% activation during right hand movement for each anatomical structure. Selected regions are displayed in inset at the left bottom of each graph (left lateral surface view, z = 4,2,6 for putamen and thalamus in Nico, Ashley and Omar respectively, and z = -21 for cerebellum). A double exponential decay function is fitted and displayed for each anatomical FC to left SM1$_{ue}$ time course (see Methods).*

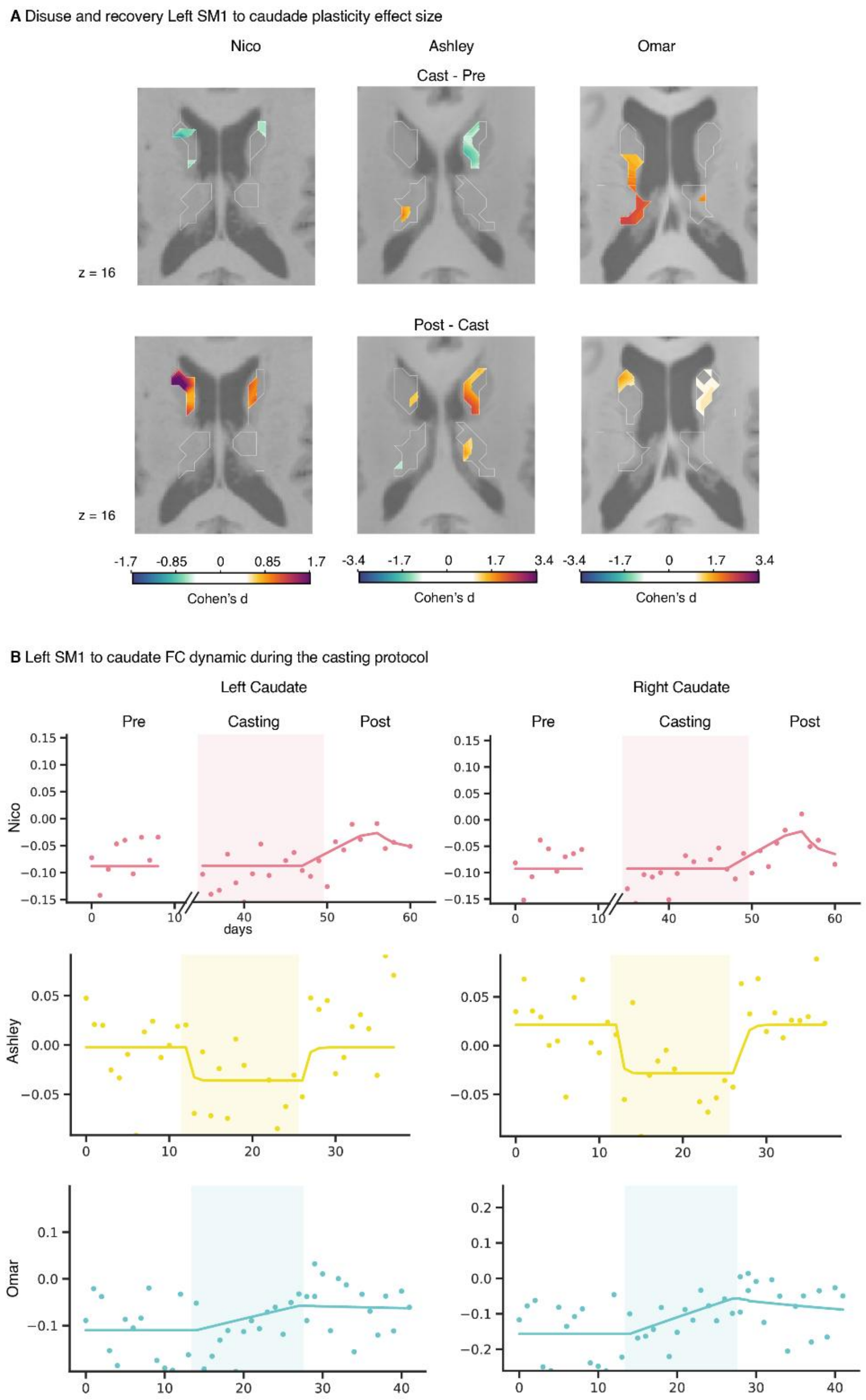

Figure S6: Disuse- and recovery-driven changes in functional connectivity (FC) of effector-specific primary somatomotor cortex (left SM1$_{ue}$) with caudate. *A) Individual-specific plasticity effect size (Cohen's d) maps showing changes in FC during (Cast - Pre) and after casting (Post - Cast) for left SM1$_{ue}$ at the z = 16 transversal MNI slice. The recovery shows a common increase in FC in caudate. However, a corresponding decrease during disuse was not detected significantly for all participants. B) FC time course over the casting protocol*

between left $SM1_{ue}$ and caudate. Regions of interest are selected as the left or right caudate voxels of significant increase after cast removal that overlap with the top 1% caudate activation during right hand movement. The time course confirms idiosyncratic FC behavior with a clear decrease and increase pattern for Ashley only.

*Note Figure S8: F*or the caudate dynamics in Nico's data, the model did not converge, as the data did not follow a clear exponential decay behavior. By allowing flexibility in the start of the decay functions, the model converges to a late decay dynamic.

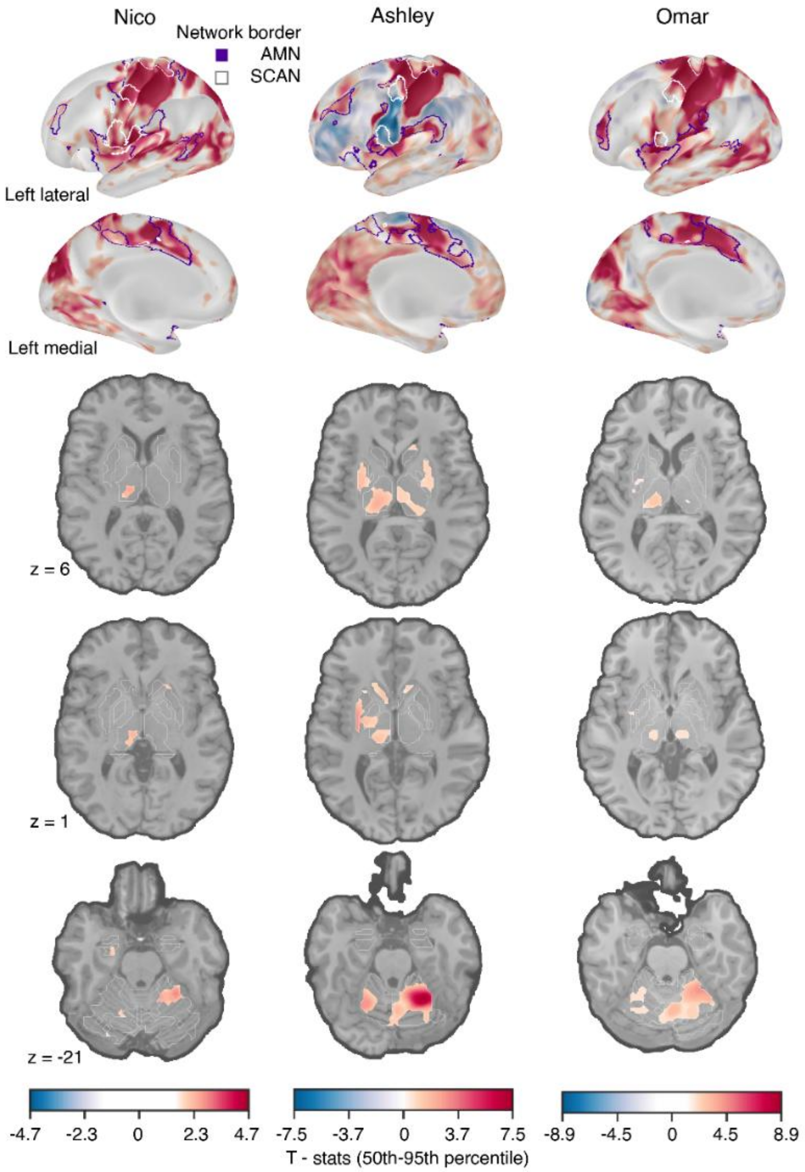

Figure S7: Spontaneous disuse pulses using standard HRF model. *T-statistics of the FSL-based standard double gamma analysis of pulse co-activation shown for the cortical left lateral and medial view and subcortical transversal slices (z= -21, 1 and 6). The Freesurfer based anatomical border of cerebellum, putamen, pallidum, thalamus, hippocampus and caudate are overlaid on the axial slices. Cortical, thalamic, and cerebellar pulse co-activations are detected within the 50th percentile of the whole brain statistics. Putamen co-activation was only detected in Ashley and Omar.*

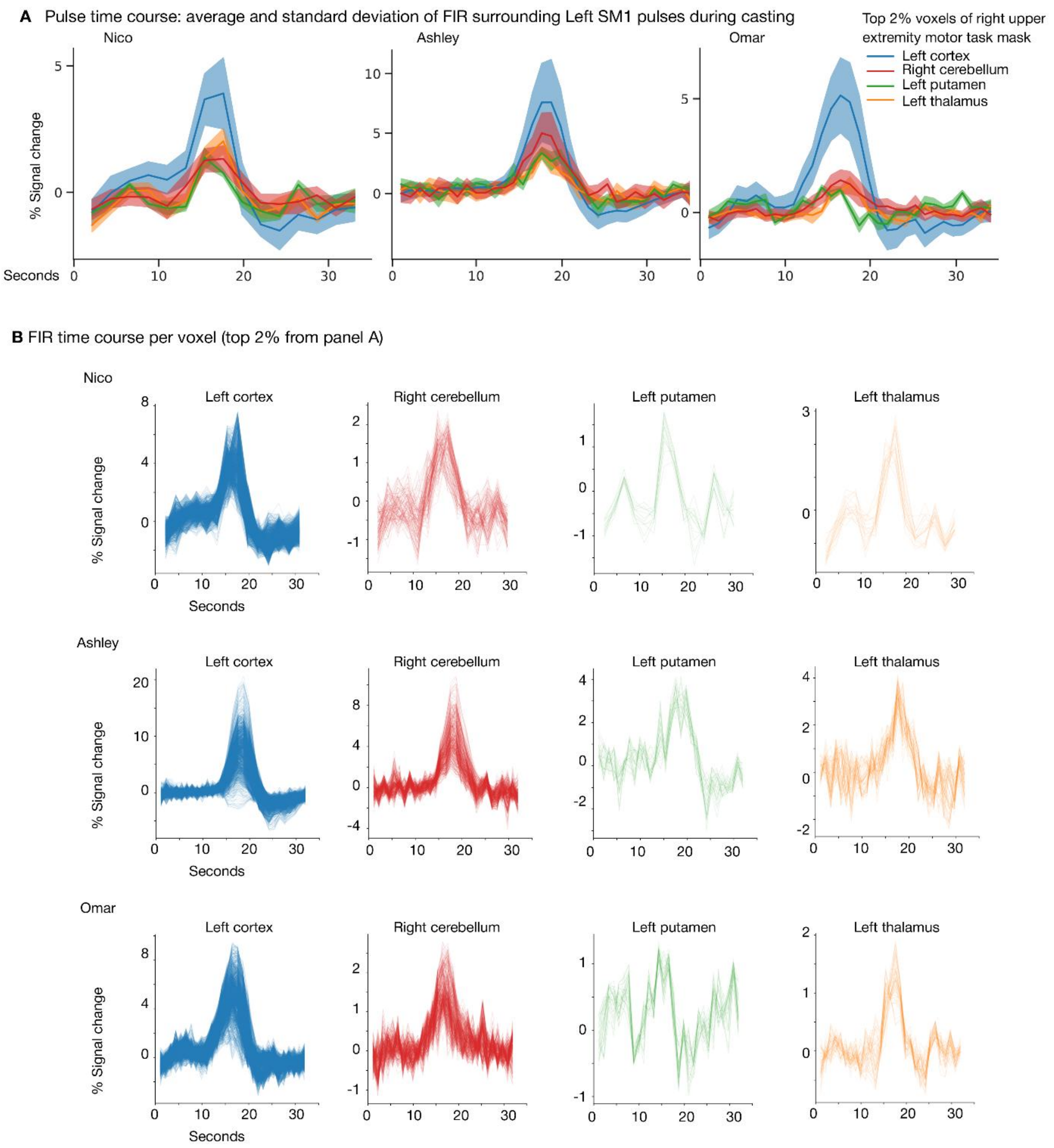

Figure S8: Spontaneous disuse pulse time courses using Finite Impulse Response (FIR) model. *Percent signal change estimated by the FIR model at each volume in a -17.6 to + 15.4 seconds window around left $SM1_{ue}$ pulse peak. A) Pulse time courses in regions of interest defined by task fMRI. For each structure (left $SM1_{ue}$, right cerebellum, left putamen and left thalamus) we selected the top 2 % voxels most strongly activated during right hand movement. The time courses (average percent signal change) are displayed during a time window (-17.6 to + 15.4 seconds around the pulse peak) surrounding the disuse pulses. B) percent signal change time course of each voxel selected in A).*

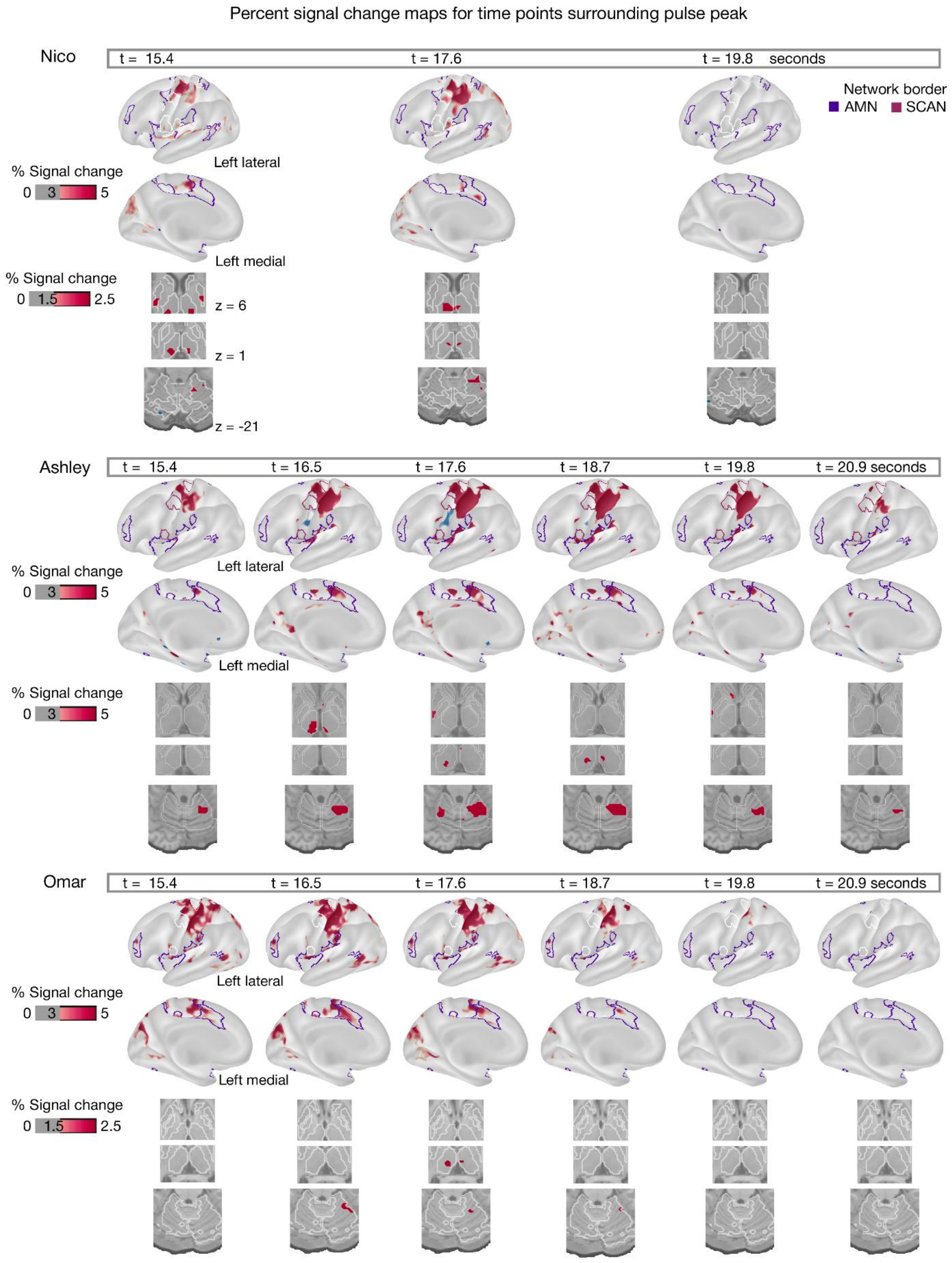

Figure S9: Spontaneous disuse pulses using Finite Impulse Response (FIR) model.
Percent signal change within +/- 3 TR of the left $SM1_{ue}$ pulse peak. The Freesurfer based

anatomical borders of the cerebellum, putamen, pallidum, thalamus, hippocampus and caudate are overlaid on the axial slices.

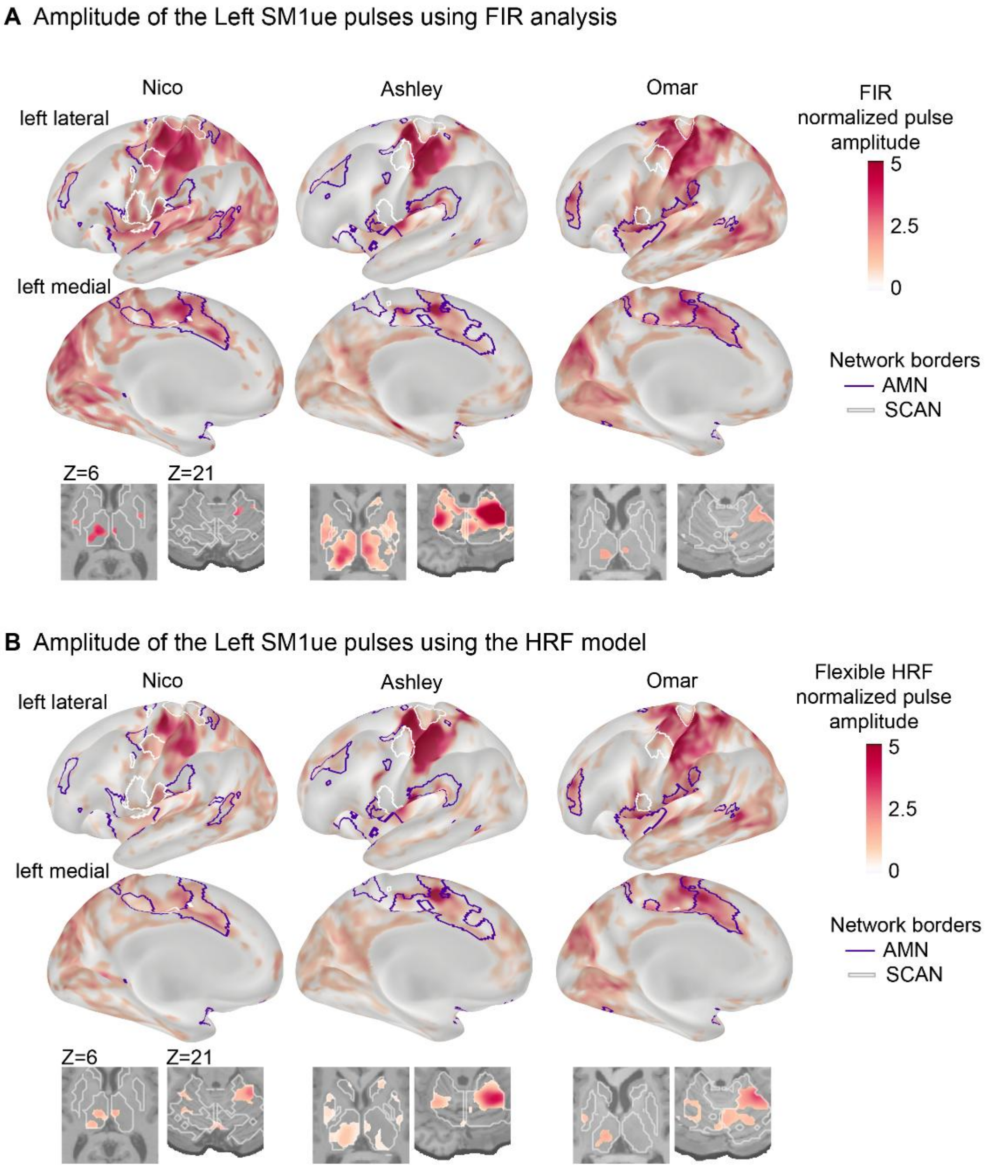

Figure S10: Spontaneous pulse amplitude maps comparison between FIR and HRF models. *A) Amplitude maps from the FIR analyses (normalized). The cortical left lateral and left medial surface are displayed with the subcortical transversal slices (z=6 and -21) for each participant (from left to right: Nico, Ashley, Omar). The Freesurfer based anatomical borders of the cerebellum, thalamus, putamen and nucleus accumbens are displayed with white borders on subcortical slices. The action-mode and somato-cognitive action network borders are displayed in purple and white on cortical surfaces. B) Amplitude maps from the HRF model. The same organization is replicated from panel A*

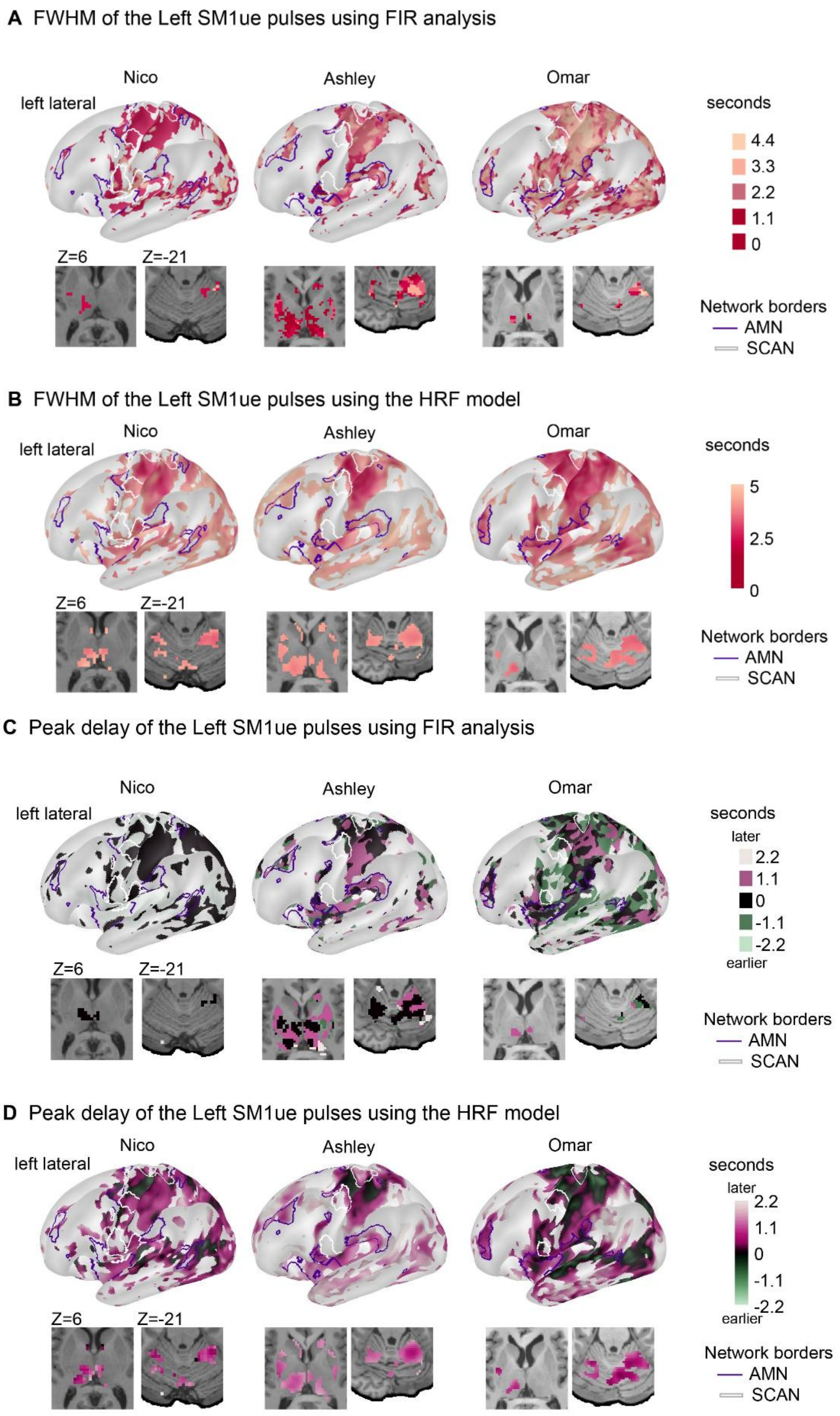

Figure S11: Spontaneous pulse full width half maximum (FWHM) and peak time delay from the left SM1$_{ue}$ region maps comparison between FIR and HRF models. *A) FWHM*

maps from the FIR analysis. The cortical left lateral and left medial surface are displayed with the subcortical transversal slices (z=6 and -21) for each participant (from left to right: Nico, Ashley, Omar. The Freesurfer based anatomical borders of the cerebellum, thalamus, putamen and nucleus accumbens are displayed with white borders on subcortical slices. The action-mode and somato-cognitive action network borders are displayed in purple and white on cortical surfaces. B) FWHM maps from the HRF model. The same organization is replicated from panel A. Due to the limited number of pulses, the FIR analysis is only able to capture discrete values. Maps are masked by positive amplitude. C) Peak delay maps from the FIR analyses D) Peak delay maps from the HRF model. The same organization is replicated from panel A. Due to the limited number of pulses, the FIR analysis is only able to capture discrete values. With a TR of 2.2seconds for Nico's fMRI acquisition, the FIR analysis fails to capture delay. Maps are masked by positive amplitude.

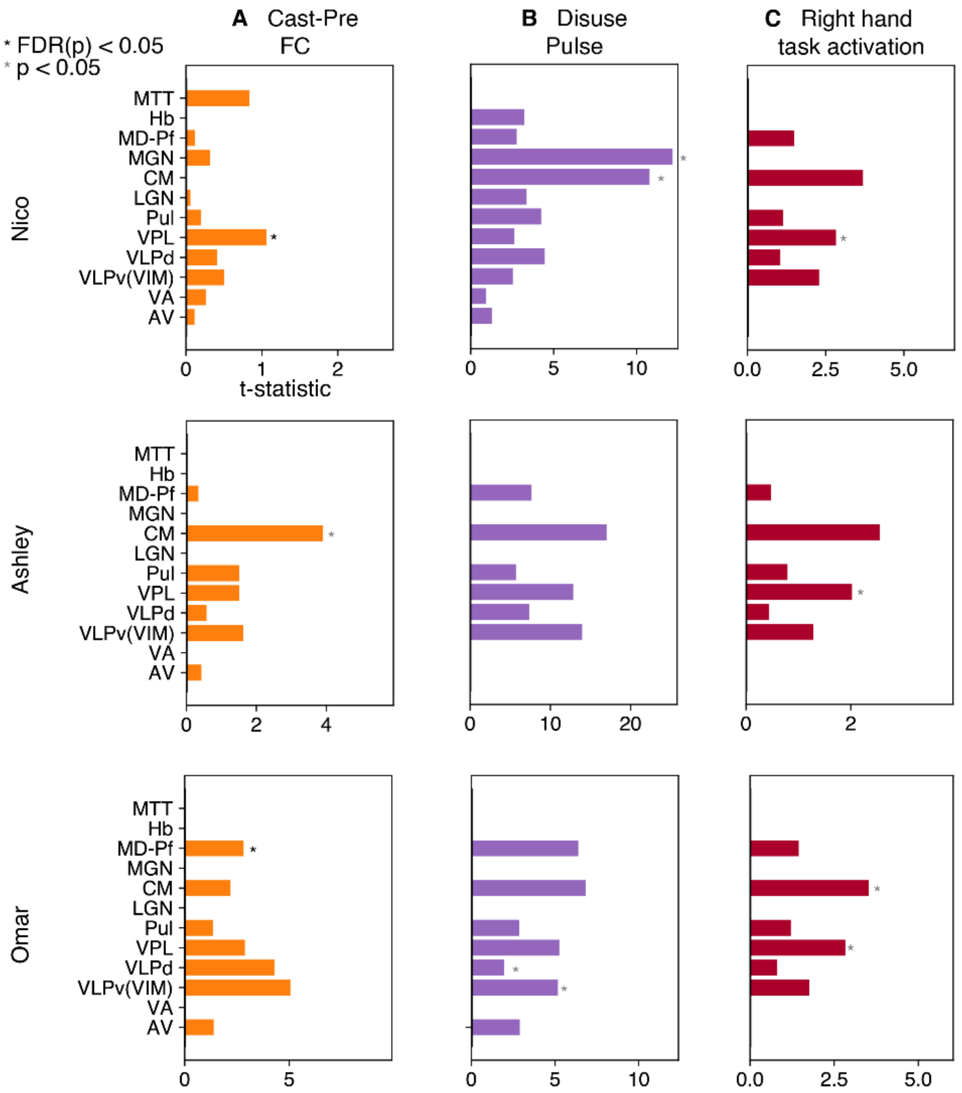

Figure S12: FC change, spontaneous pulse and motor task thalamic nuclei specificity to CM, VPL and VIM. *Average t-statistics per left thalamic nuclei of the THOMAS atlas individual segmentation (A) top 30th percentile t-statistics left thalamic map of increase FC during casting with left* $SM1_{ue}$ *region (B) top 30th percentile t-statistics left thalamic map of disuse pulses during casting (C) top 30th percentile t-statistics left thalamic map of right hand movement vs*

*baseline contrast of the HCP motor task from pre casting performance. Significance testing is performed using effect specific null distribution (see Method). * indicates p values <0.05, in black if passing false discovery rate, in gray if not. (CM: centro-median, VPL: ventro-posterior lateral, Hb: habenula, VIM: ventral intermediate, MD-Pf: medio-dorsal parafascicular, Pul: pulvinar, MGN: medial geniculate nucleus, VA: ventral anterior , MTT: mammillothalamic tract, AV: anteroventral, LGN: lateral geniculate nucleus)*

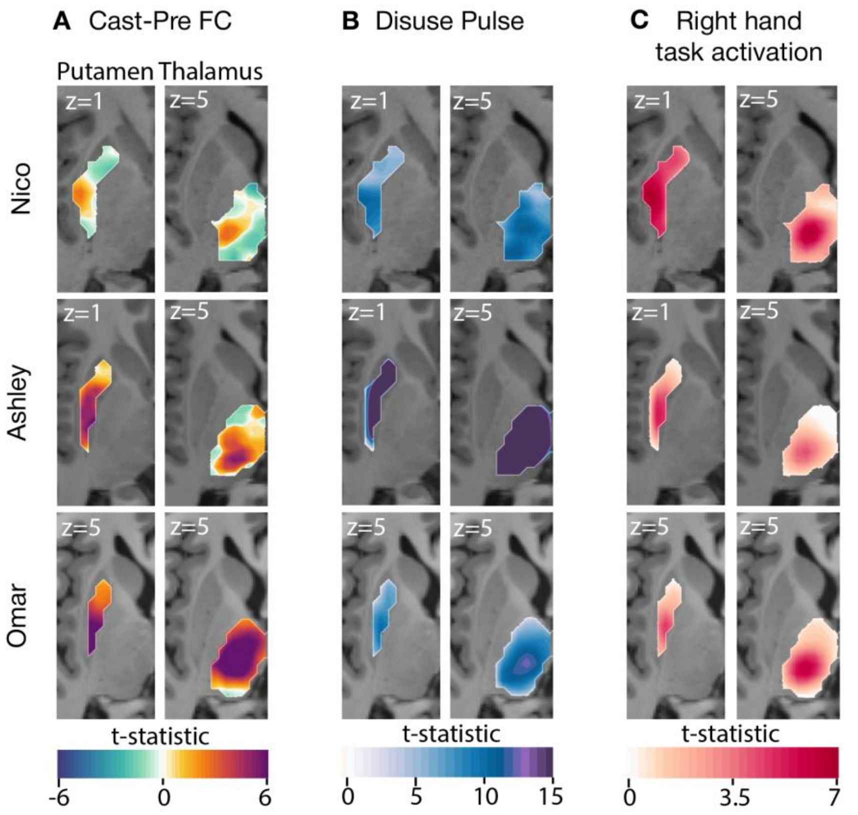

Figure S13: Putaminal and thalamic disuse-driven FC changes, pulses and hand movement task fMRI activations. *(A) Map of disuse-driven increases in FC with the left $SM1_{ue}$ region of interest. (B) Map of disuse pulses. (C) Map of pre-casting task fMRI contrast: right hand movement vs baseline. T-statistic color scales are the same across participants (from top to bottom: Nico, Ashley, Omar); unthresholded.*

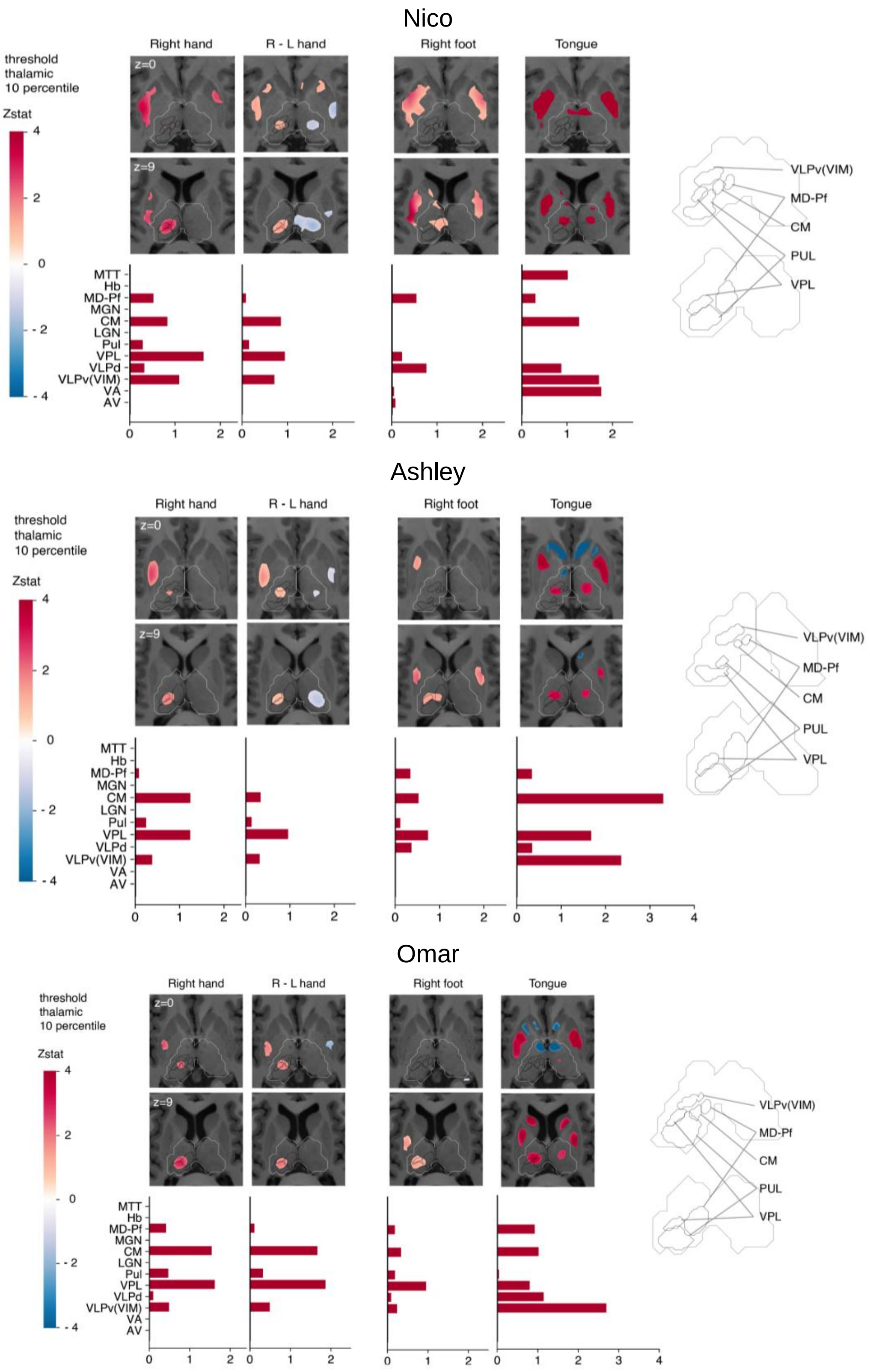

Figure S14: Subcortical representation of fMRI motor task for each participant. *Top to bottom Nico, Ashley and Omar's activation maps of HCP motor task contrast, showing the top 10 percentile relative to left thalamic activation (top) and their corresponding average per left*

thalamic nuclei (bottom). From left to right columns represent contrast of: right hand vs baseline, right - left hand, right foot, tongue. CM: centro-median , VPL: ventro-posterior lateral , Hb: habenula, VIM: ventral intermediate, MD-Pf: medio-dorsal parafascicular, Pul: pulvinar, MGN: medial geniculate nucleus, VA: ventral anterior, MTT: mammillothalamic tract, AV: anteroventral, LGN: lateral geniculate nucleus.

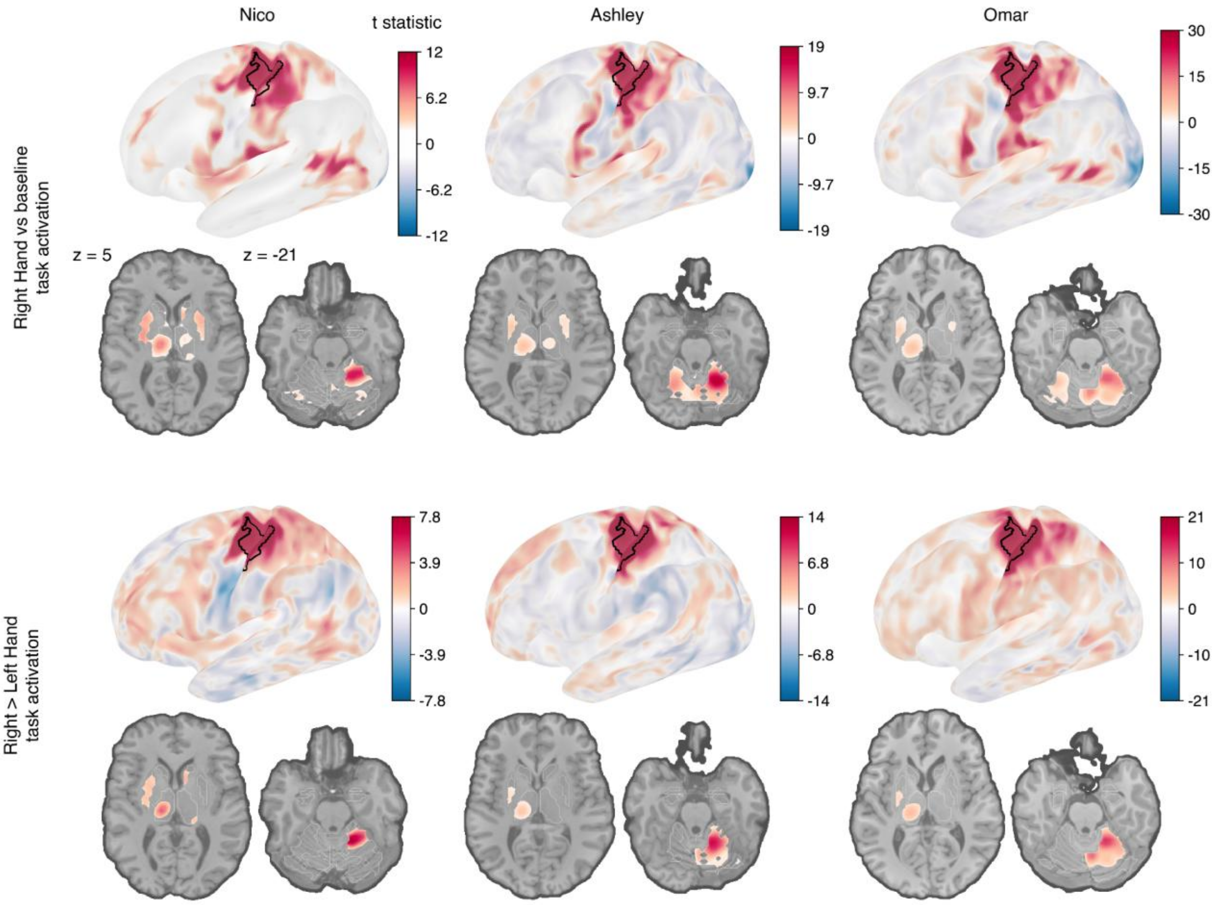

Figure S15: Right hand movement task fMRI activation maps. *T-statistic activation maps of motor tasks contrast Right hand vs baseline (top line) and Right hand > Left hand (bottom line) for each participant. Maps are thresholded to show the top 30% most activated voxels. The black border indicates the hand primary motor cortex area for reference on the inflated surface. Axial slices z=5 and z=-21 highlight the main activations in putamen, thalamus and cerebellum respectively.*

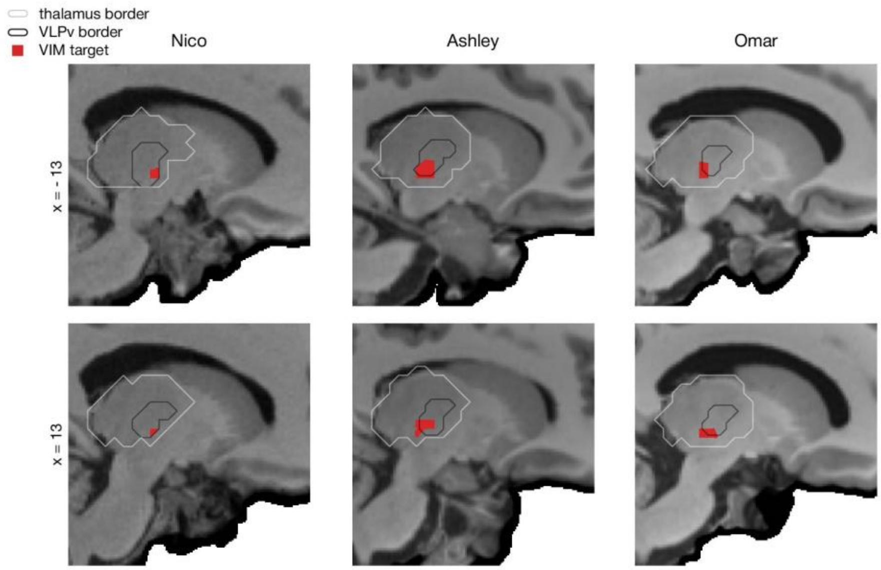

Figure S16: Localization of thalamic VIM (ventral-intermediate) target. *The union of possible VIM target voxels (red) within the 2 mm anterior and 3 mm superior of the ACPC targeting formula. For each participant (Nico, right; Ashley, center; Omar, left), and both hemisphere (Left, top; Right, bottom), the THOMAS segmentation border of the Ventrolateral Posterior ventral nuclei (VLPv; black) and thalamic (white) borders are overlaid to highlight the overlap of VIM (target to descend stimulation electrode) with the bottom of the VLPv.*

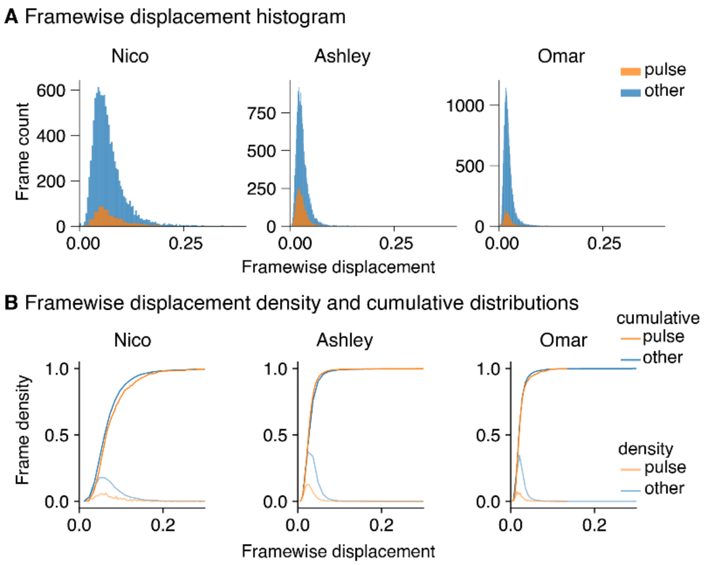

Figure S17: Head movement during pulses. *A) Histograms of framewise displacement values (FD in mm) for frames with pulses (orange) and frames without pulses (blue), for all three participants (Nico, Ashley, Omar). B) Density and cumulative distributions of framewise displacement values (FD in mm) for frames with pulses (orange) and frames without pulses (blue), for all three participants (Nico, Ashley, Omar). There is no reproducible difference in head movement between pulse and non-pulse frames (two-sample Kolmogorov-Smirnov tests, at p=0.005: Nico pulse FD< without pulse FD, Ashley pulse FD > without pulse FD, Omar no significant difference).*
